# Minimum complexity drives regulatory logic in Boolean models of living systems

**DOI:** 10.1093/pnasnexus/pgac017

**Published:** 2022-04-15

**Authors:** Ajay Subbaroyan, Olivier C Martin, Areejit Samal

**Affiliations:** The Institute of Mathematical Sciences (IMSc), Chennai 600113, India; Homi Bhabha National Institute (HBNI), Mumbai 400094, India; Université Paris-Saclay, CNRS, INRAE, Univ Evry, Institute of Plant Sciences Paris-Saclay (IPS2), 91405 Orsay, France; Université de Paris, CNRS, INRAE, Institute of Plant Sciences Paris-Saclay (IPS2), 91405 Orsay, France; The Institute of Mathematical Sciences (IMSc), Chennai 600113, India; Homi Bhabha National Institute (HBNI), Mumbai 400094, India

**Keywords:** gene regulatory networks, Boolean networks, update rules, Boolean complexity, average sensitivity

## Abstract

The properties of random Boolean networks have been investigated extensively as models of regulation in biological systems. However, the Boolean functions (BFs) specifying the associated logical update rules should not be expected to be random. In this contribution, we focus on *biologically meaningful* types of BFs, and perform a systematic study of their preponderance in a compilation of 2,687 functions extracted from published models. A surprising feature is that most of these BFs have odd “bias”, that is they produce “on” outputs for a total number of input combinations that is odd. Upon further analysis, we are able to explain this observation, along with the enrichment of read-once functions (RoFs) and its nested canalyzing functions (NCFs) subset, in terms of 2 complexity measures: *Boolean complexity* based on string lengths in formal logic, which is yet unexplored in biological contexts, and the so-called *average sensitivity*. RoFs minimize Boolean complexity and all such functions have odd bias. Furthermore, NCFs minimize not only the Boolean complexity but also the average sensitivity. These results reveal the importance of minimum complexity in the regulatory logic of biological networks.

Significance Statement Regulatory rules arising in biological networks are expected to be far from random. To validate this expectation, we introduce a quantitative framework and perform detailed analyses of a dataset of 2,687 BFs compiled from 88 reconstructed discrete logical models of biological systems. Our approach reveals that in fact regulatory rules preferentially minimize complexity defined via either Boolean complexity or average sensitivity, 2 complexity measures that are strongly correlated. In particular, this second complexity measure is minimized by NCFs, a class of rules that are commonly used when modeling gene regulatory networks.

## Introduction

Cells are the building blocks of all living organisms and their decision-making is tightly controlled by complex and intricate gene regulatory networks (1). Much work over the past 3 decades has led to a deeper understanding of the structure and dynamics of these complex biological networks (2–9). One of the most useful frameworks for probing the *dynamical* aspects of such networks is the so-called “logical modeling” approach first introduced by Stuart Kauffman (10) and René Thomas (11). In its usual formulation, it assumes a Boolean simplification in which all biological entities are taken to be “on” or “off”. Kauffman considered ensembles of such Boolean networks in which the input–output rules were chosen at random (12), an idealization allowing the characterization of the attractors in these networks (2, 13, 14).

Extensive studies of biological networks made possible by recent advances in large-scale data acquisition have revealed that their topological structure is very far from random (5, 6, 9, 15). Furthermore, various Boolean dynamical models of such systems (16–21) have been constructed in the last 2 decades. It is now important to characterize the properties of the Boolean functions (BFs) encoding the associated regulatory rules to distinguish them from randomly chosen functions. In previous studies (2, 22, 23), 1 property that has been used to characterize BFs is the fraction of occurrences of the output value “1” when considering all possible combinations of input values. Feldman (24) proposed a way to classify BFs using the number (*k*) of inputs to the BF and the number of occurrences of the output value “1”, which we refer to as the bias *P*. One can also consider more functional aspects of the BFs, leading to what can be called *biologically meaningful* types of BFs. In this work, we systematically study different types of biologically meaningful BFs and their occurrence both in the complete space of $2^{2^k}$ BFs for a *k*-input BF, and in a reference biological dataset of 2,687 BFs compiled from 88 published biological models.

Kauffman (2) had proposed that the occurrence of logical rules could be shaped by the constraint of being “chemically simple”. Here, we borrow concepts from the computer science literature to quantify the notion of simplicity (or complexity) of a BF and then perform a thorough evaluation of the biologically meaningful types of BFs from the perspective of complexity. The 2 measures of complexity which we exploit are Boolean complexity (24) and average sensitivity (22, 25). We show that read-once functions (RoFs) (26) that constitute all logical rules with minimal Boolean complexity are highly over-represented in the biological data. Further, we provide an analytical proof that nested canalyzing functions (NCFs) (19), which are a subset of RoFs, minimize not only the Boolean complexity but also the average sensitivity across all BFs in Feldman’s associated *k*[*P*] set. Our result that NCFs are minimally complex in terms of both complexity measures is a likely explanation for their prevalence in biological data. In a nutshell, our exploration of 2 complexity measures using 2,687 BFs compiled from published models puts Kauffman’s conjecture of “preference for simplicity” on a sound footing while refining it, using a quantitative framework for rule complexity in gene regulatory networks.

## Background

### Boolean models of biological networks

A Boolean model of a biological system consists of a network of *N* nodes and *L* edges, wherein the nodes correspond to components such as genes or proteins and (directed) edges capture the regulation of 1 node by a set of other nodes (2, 10–12). Let us label each node of the network by an integer *i* (*i* = 1,..., *N*) and denote the “on” or “off” state of node *i* by a Boolean variable *x*_*i*_ ∈ {0, 1}. The state *x*_*i*_, output by node *i* in the Boolean model, is determined by: (a) the values of its *k*_*i*_ inputs, coming from the *k*_*i*_ nodes from which it has incoming links, and (b) a logical update rule or *Boolean functionf*_*i*_ that specifies how *x*_*i*_ changes in time or is *updated* given those *k*_*i*_ inputs. (a) and (b) along with an update scheme over the different nodes (*synchronous* (2) or *asynchronous* (11, 27)) determine the dynamics of the Boolean network. The different representations of BFs relevant to this work are given in Section 1 and Figure S1 in Supplementary Material.

### Categorization of BFs based on their bias and use of isomorphisms

Feldman (24, 28) grouped BFs based on their number of input variables (*k*) and bias (*P*). The bias *P* of a BF is the number of 1s in the output column of its truth table (see Figure S1, Supplementary Material). The BFs with a given *k* and *P* constitute the *k*[*P*] set. Evidently, the number of *k*[*P*] sets for a given *k* is 2^*k*^ + 1. Note that every function in *k*[*P*] has a *complementary* function in *k*[2^*k*^ − *P*] obtained via complementation of the corresponding Boolean expression (28) where “on” and “off” states are exchanged.

Within any given *k*[*P*] set, Feldman (24, 28) introduced a partitioning into equivalence classes based on isomorphisms. Two BFs *f* and *g* are defined as *isomorphic* if they are identical up to permutations and negations of any of their input variables. For example, the BF *f* = *x*_1_ · (*x*_2_ + *x*_3_) is isomorphic to the BF $g =x_2\cdot (x_1 + \overline{x}_{3})$. For our work, we associate 1 “representative” BF to each class, specifically the one in which the first occurrence of each variable arises both sequentially (with indices 1, 2, 3, …) and as a positive literal. Interestingly, Reichhardt and Bassler (29), using concepts borrowed from chemistry and group theory, have shown how to enumerate the distinct isomorphic classes in each *k*[*P*] set.

We describe some properties associated with the bias of a BF obtained by combining 2 independent BFs in Section 2 in Supplementary Material.

## Complexity Measures

Various measures of *complexity* of BFs have been studied in the computer science literature (25, 30, 31). We adopt 2 of them in this work, namely, Boolean complexity and average sensitivity.

### Minimal expressions and Boolean complexity

The first measure of complexity we use, formulated in particular by Feldman (24), is called the Boolean complexity. In principle, there are an infinite number of logical expressions corresponding to a given BF (24, 30). Feldman (24) focused on the shortest possible expression when considering the number of literals it is composed of, the so called *minimal formula* for a BF. Feldman defined the *Boolean complexity* of a BF to be the number of literals in its minimal formula (24, 30). Though Boolean expression types such as the minimal canonical disjunctive normal form (DNF) or the minimal canonical conjunctive normal form (CNF) are widely used to represent BFs, they are typically distinct from the minimal formula as defined by Feldman (24).

For instance, the 3-input BF in the minimal canonical DNF, $f(x_{1},x_{2},x_{3}) = \overline{x}_{1} \overline{x}_{2} \overline{x}_{3} + \overline{x}_{1} \overline{x}_{2} x_{3} + \overline{x}_{1} x_{2} \overline{x}_{3}$ containing 9 literals can be shown to be equivalent to a minimum formula containing 3 literals by applying the laws of Boolean algebra as follows:

\begin{eqnarray*} f(x_{1},x_{2},x_{3}) &=& \overline{x}_{1} \overline{x}_{2} \overline{x}_{3} + \overline{x}_{1} \overline{x}_{2} x_{3} + \overline{x}_{1} x_{2} \overline{x}_{3} \\ &=& \overline{x}_{1}(\overline{x}_{2} \overline{x}_{3} + \overline{x}_{2} x_{3} + x_{2} \overline{x}_{3}) \\ &=& \overline{x}_{1}(\overline{x}_{2} (\overline{x}_{3} + x_{3}) + x_{2} \overline{x}_{3}) = \overline{x}_{1}(\overline{x}_{2} + x_{2} \overline{x}_{3}) \\ &=& \overline{x}_{1}((\overline{x}_{2} + x_{2})(\overline{x}_{2} + \overline{x}_{3})) = \overline{x}_{1} (\overline{x}_{2}+\overline{x}_{3}). \end{eqnarray*}

Here, *x*_*i*_ and $\overline{x}_{i}$ represent a positive and negative literal, respectively. In the above simplification, we employ the law $\overline{x} + x = 1$, and the distribution property over the OR (+) operator. Thus, the minimal irreducible expression $f(x_{1},x_{2},x_{3}) = \overline{x}_{1}(\overline{x}_{2} + \overline{x}_{3})$ has 3 literals and the function has Boolean complexity equal to 3. However, note that the minimal DNF for this BF is $\overline{x}_{1} \overline{x}_{2} + \overline{x}_{1} \overline{x}_{3}$, which has 4 literals, and factorization of this expression is necessary to obtain the minimal expression with 3 literals for the above BF.

#### Computing the Boolean complexity

Obtaining a minimal formula for a given BF or expression is a computationally hard problem (32). In practice, one has to resort to heuristic algorithms such as the QMV proposed by Vigo (33) for reducing expressions. Thus, barring exceptions, one can only obtain an upper bound on the Boolean complexity for BFs with several inputs. In our work, to obtain the factorized minimal expression of a BF, we employ the logic synthesis software “ABC” (34, 35). To improve the estimated Boolean complexity of a BF, we give as input to the ABC software 4 types of Boolean expressions, namely the full DNF, the full CNF, the Quine–McCluskey minimized DNF expression (36, 37), and the Quine–McCluskey minimized CNF expression, corresponding to the same BF. As a result, 4 output Boolean expressions are obtained of which the one with the least number of literals is chosen as the minimal equivalent expression of the BF. The number of literals in this expression is then our estimate of the Boolean complexity of that BF.

### Average sensitivity of BFs

The second measure of complexity we use, the average sensitivity, is based on how sensitive a BF is to changes of its inputs (22). For a BF *f* with *k*-inputs, the *sensitivity* for a given assignment of the input variables $\mathbf {x}=(x_1=a_1,x_2=a_2,\ldots ,x_k=a_k)$ is the number of *neighbors*$\mathbf {y}$ of $\mathbf {x}$ for which the output $f(\mathbf {y})$ is different from $f(\mathbf {x})$ (22, 25). The assignments $\mathbf {y}$ and $\mathbf {x}$ are “neighbors” if they differ in exactly 1 of their *k* variables. The average of the sensitivity over all input combinations gives the average sensitivity of a BF, and is given by the expression:

(1) \begin{eqnarray*} S_f = \left\langle \sum _{i=1}^{k} f(\mathbf {x} \oplus \mathbf {e}_{i}) \oplus f(\mathbf {x}) \right \rangle _\mathbf {x}, \end{eqnarray*}

where ⊕ is the XOR operator and $\mathbf {e}_{i} \in \lbrace 0, 1\rbrace ^{k}$ denotes the unit vector corresponding to having input variable *x*_*i*_ = 1 and all other input variables set to 0. $\mathbf {x}$ can be mapped to a vertex *V* of a *k*-dimensional Boolean hypercube (or *k*-cube). The sensitivity at $\mathbf {x}$ then has a geometric interpretation: it is the number (between 0 and *k*) of neighbors of *V* whose output value differs from that of *V*. The total sensitivity of *f*, which is the sum of the sensitivities over all the vertices of the *k*-cube is equal to twice the number of *k*-cube edges whose 2 ends are vertices with complementary output values. It follows from the above definition that the lower the average sensitivity of a BF, the more *robust* it is to changes of its input variables (22).

Note that isomorphic BFs have identical average sensitivities. Indeed, the operations of rotations or reflections about any of the axes of the hypercube do not change the number of “red” and “blue” neighbors with output values 1 or 0, respectively, for any vertex (see Figure S1(d), Supplementary Material). Moreover, a BF and its complement belonging to sets *k*[*P*] and *k*[2^*k*^ − *P*], respectively, also have the same average sensitivity. This is because under complementation of the BF, the “red” and “blue” vertices of the *k*-cube are exchanged, thereby leaving the number of edges *E*_01_ in the *k*-cube unchanged (see Figure S1(d), Supplementary Material).

## Biologically Meaningful Types of BFs

The number of BFs having *k*-inputs is $2^{2^{k}}$ (see Section 1 in Supplementary Material). Clearly, this number explodes with growing number of inputs (Figure S2 and Table S1, Supplementary Material). It is, thus useful to focus on those subsets of BFs which possess *biologically meaningful* properties (38). Here, we describe some of the biologically meaningful BFs and give their important properties whose proofs are provided in Section 3 in Supplementary Material.

### Effective function (EF)

A regulatory input is called *effective* if and only if there exists some input condition, wherein the modulation of that input alters the output of the considered function. If such a condition does not exist, that regulator (or input) can be considered to be *ineffective*. It follows that all inputs of a biological BF ought to be effective (38): if an input is ineffective, it should not be counted as a regulatory input. Formally, a BF *f* with *k*-inputs is an Effective function (EF) iff:

(2) \begin{eqnarray*} \forall \ i \in [1,k],\ \exists \ \mathbf {x} \in \lbrace 0, 1\rbrace ^{k}\ \text{with}\ x_{i} = 0, \ f(\mathbf {x}) \ne f(\mathbf {x} + \mathbf {e}_{i}), \end{eqnarray*}

where $\mathbf {e}_{i} \in \lbrace 0, 1\rbrace ^{k}$ denotes the unit vector associated to the component of index *i*. We find that all ineffective BFs have even bias (see Property 3.1 in Supplementary Material). Furthermore, a *k*-input EF possesses a Boolean complexity that is at least *k* (see Property 3.2 in Supplementary Material).

### Unate function (UF)

A regulatory element may activate or inhibit the expression of a target gene. Such activatory/inhibitory relationships can be formalized as follows (39): a BF *f* with *k*-inputs is said to be activating (increasing monotone) in its input *i* (or variable *x*_*i*_) iff:

(3) \begin{eqnarray*} \forall \ \mathbf {x} \in \lbrace 0, 1\rbrace ^{k}\ \text{with}\ x_{i} = 0, \, f(\mathbf {x}) \le f(\mathbf {x} + \mathbf {e}_{i}), \end{eqnarray*}

and inhibiting (decreasing monotone) in its input *i* (or variable *x*_*i*_) iff:

(4) \begin{eqnarray*} \forall \ \mathbf {x} \in \lbrace 0, 1\rbrace ^{k}\ \text{with}\ x_{i} = 0, \, f(\mathbf {x}) \ge f(\mathbf {x} + \mathbf {e}_{i}). \end{eqnarray*}

A BF *f* with *k*-inputs is said to be a sign-definite or *unate function* (UF) iff each input *i* = 1, 2, …, *k* is either activating or inhibiting (39). For further classification of UFs into different combinations of activating and inhibiting inputs, see Figure S3 (Supplementary Material). We now list some properties of UFs which we utilize in this work. UFs can be represented by a DNF expression in which all occurrences of any specific input variable (more precisely, literal) are either negated (i.e. negative input) or non-negated (i.e. positive input) (39, 40) (see Property 3.3 in Supplementary Material). If *u*_1_ and *u*_2_ are UFs with *k*_1_ and *k*_2_ independent input variables, respectively, then the combined BF *u* = *u*_1_⊙*u*_2_, where ⊙ ∈ {∧, ∨}, is also unate (see Property 3.4 in Supplementary Material). Here, ∧ and ∨ are the AND and OR operators respectively. If an input *i* of a UF *u* acts as both an activator and an inhibitor, then input *i* is ineffective (see Property 3.5 in Supplementary Material).

### Canalyzing function (CF)

A BF *f* with *k*-inputs is said to be canalyzing in an input *i* (or variable *x*_*i*_) if and only if

(5) \begin{eqnarray*} f(x_{1}, x_{2}, \ldots ,x_{i-1},x_i=a,x_{i+1},\ldots ,x_{k})=b, \end{eqnarray*}

independent of *x*_*j*_ for *j* ≠ *i*. In the above equation, *a* and *b* can take values 0 or 1, *a* is the canalyzing input value and *b* is the canalyzed value for input *i*. A BF *f* is a *canalyzing function* (CF) if at least 1 of its *k*-inputs satisfies the canalyzing property (2).

### Nested canalyzing function (NCF)

NCFs have been previously studied in several works, see e.g. (19, 41, 42). A NCF with *k*-inputs can be represented as a Boolean expression with exactly *k* literals as follows (42, 43):

(6) \begin{eqnarray*} f(\mathbf {x}) = X_{\sigma (1)} \odot ( X_{\sigma (2)} \odot ( X_{\sigma (3)} \odot \ldots ( X_{\sigma (k-1)} \odot X_{\sigma (k)})))\,, \end{eqnarray*}

where σ is a permutation on the inputs {1, 2, …, *k*}, *X*_σ(*i*)_ ∈ {*x*_σ(*i*)_, $\overline{x}_{\sigma (i)}\rbrace$ and ⊙ ∈ {∧, ∨}. NCFs have odd bias, are effective, are unate, and have Boolean complexity equal to the number of inputs *k* (see Properties 3.6, 3.7, 3.8, and 3.9, respectively in Supplementary Material).

### Read-once function (RoF)

A BF of *k* variables is a RoF if it can be represented by a Boolean expression, using the operations of conjunction, disjunction and negation, in which every variable appears exactly once (26). Mathematically, a *k*-input BF *f* is a RoF iff there is a permutation σ on {1, 2, …, *k*} such that, after stripping of parentheses, *f* can be written as

(7) \begin{eqnarray*} f(\mathbf {x}) = X_{\sigma (1)} \odot X_{\sigma (2)} \odot X_{\sigma (3)} \ldots \odot X_{\sigma (k)}, \end{eqnarray*}

where as before *X*_σ(*i*)_ ∈ {*x*_σ(*i*)_, $\overline{x}_{\sigma (i)}\rbrace$ and ⊙ ∈ {∧, ∨}. The formula for the RoF requires including the parentheses but there are no restrictions on where these are placed. For example, the expressions *x*_1_*x*_2_(*x*_3_ + *x*_4_) and *x*_1_(*x*_2_*x*_3_ + *x*_4_) correspond to distinct RoFs. All *k*-input RoFs can be generated recursively by pairing *i*-input RoFs with *j*-input RoFs such that *i* + *j* = *k* (see Property 3.10 in Supplementary Material). RoFs have odd bias, are effective, are unate, and have the lowest Boolean complexity among all EFs for a given number of inputs *k* (see Properties 3.11, 3.12, 3.13, and 3.14, respectively in Supplementary Material). Note that NCFs form a subset of RoFs (see Property 3.15 in Supplementary Material). Interestingly, we show that any RoF with bias *P* = 1,  3,  or  5, regardless of *k*, is always an NCF (see Property 3.16 and Figure S4 in Supplementary Material). Using these properties, we generated a catalog and provide a procedure to check whether a BF is a RoF for *k* ≤ 10 (see Section 4 and Figure S5 in Supplementary Material).

## Characterizing the Overlapping Sets of Biologically Meaningful BFs

We now can systematically explore the relationships between the aforementioned types of biologically meaningful BFs. To the best of our knowledge, such a combined delineation of the different types of biologically meaningful BFs in the space of all $2^{2^k}$ BFs has not been carried out previously. Exhaustive enumeration of BFs for low values of *k* led us to conjecture some properties of these BFs for which we provide analytical proofs (see Section 3 in Supplementary Material).

**Fig. 1:**
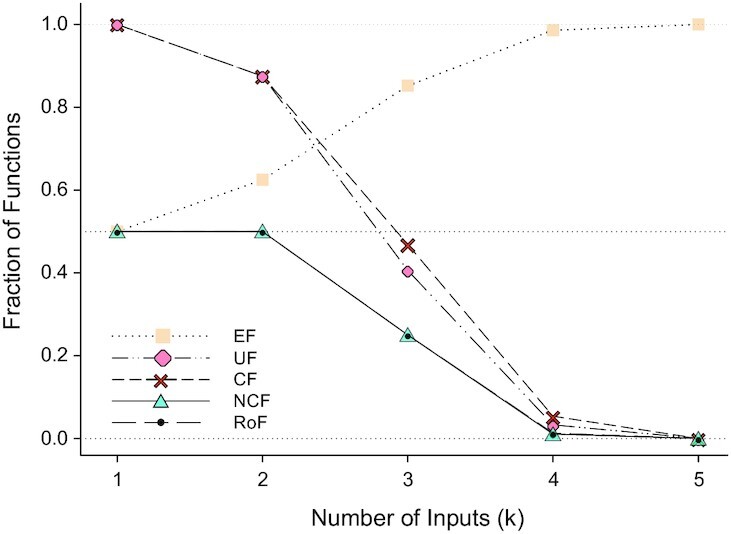
The fraction of biologically meaningful types of BFs among all BFs for a given number of inputs *k* ≤ 5.

Computational enumeration up to *k* ≤ 5, shows that the fraction of EFs in the space of all *k*-input BFs increases with increasing *k*. In contrast, the fraction of UFs and CFs decreases with increasing *k* and tend to 0 (see Fig. 1 and Table S2, Supplementary Material). The proportions of even bias functions within the sets EFs, UFs and CFs and also in their intersections at *k* ≤ 5 seem to tend to 0.5 for increasing *k* (see Table S3, Supplementary Material). Note that for a given number of inputs but various combinations of activators and inhibitors the proportion of even bias functions is constant (see Table S4, Supplementary Material). Furthermore, computational enumeration up to *k* ≤ 10, shows that the fraction of RoFs, NCFs and non-NCF RoFs among all BFs with exactly *k*-inputs decreases and tends to 0 with increasing *k* (see Fig. 1 and Table S5, Supplementary Material). We find that in the set of RoFs for a given value of *k*, the fraction of these functions that are also NCFs decreases with increasing *k* (see Table S5, Supplementary Material). It is also feasible to perform such enumerations separately for the different possible values of the bias *P*. In Figure S4 (Supplementary Material), we show the corresponding numbers for RoFs, NCFs, and non-NCF RoFs with *k* = 4, 5, 6, 7,  and  8.

**Fig. 2:**
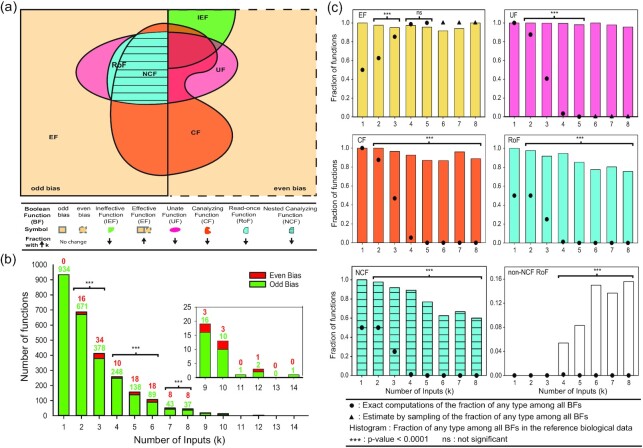
(a) In the space of all BFs, a schematic of the overlaps between different types of biologically meaningful BFs with 4 inputs. This figure is not drawn to scale but the sizes of the sets corresponding to different types of BFs and their intersections respect the order of the actual values. The legend gives the correspondence between shapes with specific color and the different types of BFs. Ordering the different types of BFs with 4 inputs (which are *not* mutually exclusive) based on their sizes in a descending order gives: EF > Odd bias = Even bias > CF > UF > RoF > NCF. The up (or down) arrows in the legend depict the increase (or decrease) in the fraction of BFs that belong to a specific type as *k* increases (see Table S2 (Supplementary Material) for the exact numbers). (b) The in-degree distribution for nodes in the reference biological dataset. (c) The plots show the abundance and statistical significance of the biologically meaningful BFs for *k* ≤ 8 in the reference biological dataset. The dot symbols which appear to coincide with the *x*-axis are very small nonzero numbers (except for non-NCF RoFs with *k* = 1, 2, and 3).

Figure 2(a) gives an overview of the space of biologically meaningful BFs across all 4-input BFs and serves as a visual guide to the overlaps between the different types of BFs. The space of all BFs can be divided into 2 equal parts based on the parity (odd and even) of the bias. Interestingly, all ineffective BFs (IEFs) lie in the even bias half. This raises the question as to whether all IEFs have even bias. We theoretically prove that this is indeed the case (see Property 3.1 in Supplementary Material). The UFs, which allow for all possible numbers of activators and inhibitors, are rather evenly distributed across even and odd biases and have some overlap with the IEF set (Fig. 2(a)). Indeed, not all UFs are EFs (see Property 3.5 in Supplementary Material). The CFs, like the UFs, are almost equally distributed across even and odd biases and overlap with the IEFs, EFs, and UFs (Fig. 2(a)).

Next, RoFs lie in the odd bias half (Fig. 2(a)). This warrants the conjecture that all RoFs have odd bias, and we show that this is indeed the case (see Property 3.11 in Supplementary Material). Moving to the NCFs, we see in Fig. 2(a) that NCFs lie within the space of RoFs (see Property 3.15 in Supplementary Material). Thus, NCFs also have odd bias. NCFs are also a strict subset of RoFs when *k* ≥ 4. For a proof, see Property 3.6 in Supplementary Material.

## Enrichments in the Biological Data

In this section, we report on the relative abundance and associated statistical significance of the different types of BFs in a compiled dataset of 2,687 BFs from 88 reconstructed models. For details on the compiled reference biological dataset and the statistical tests carried out, see Section 5 and Section 6, respectively in Supplementary Material. The in-degree distribution for these 2,687 BFs, represented in Fig. 2(b), shows that the number of these BFs decreases rapidly with increasing *k* (Fig. 2(b)). The key methodology, hereafter consists in focusing on the relative abundances of the different types of BFs when comparing the ensemble of all BFs to the ensemble composed of our reference dataset. A statistically significant enrichment is suggestive of some selection pressure on the BFs in the biological networks.

### Enrichment in types when comparing to the ensemble of random BFs

Figure 2(b) indicates that for in-degrees 1 ≤ *k* ≤ 8, the odd bias BFs are dominant and statistically enriched in the reference dataset. It is not immediately apparent why BFs with odd bias should be preferred over BFs with even bias as biologically meaningful BFs with even bias do exist, e.g. a subset of functions, which are both unate and canalyzing can have even bias (see Fig. 2(a)). Furthermore, among 2-input BFs, the XOR and XNOR functions have even bias but are completely absent from our reference biological dataset.

Figure 2(c) shows the relative abundances, in the reference dataset (see Tables S6 and S7 (Supplementary Material) for exact values) and in the ensemble of random BFs, of the various types of BFs. Statistical tests reveal that the relative abundances in the reference biological dataset are larger (1-sided *p*-values) than those expected under the null hypothesis, whereby the reference BFs are drawn from the ensemble of random BFs (see stars above the bars in Fig. 2 and Table S8 (Supplementary Material) for *p*-values), with the exception of the EFs. This exception is justified by the fact that random functions are typically EFs (see Fig. 1). The ratios provided in Table 1 show that the RoF, NCF, and the non-NCF RoF types are all strongly enriched in the reference dataset.

**Table 1.** Fractions of functions that are RoFs, non-NCF RoFs, or NCFs, in the space of all $2^{2^{k}}$BFs (*f*_0_) or in the reference biological dataset (*f*_1_). *E*( = *f*_1_/*f*_0_) is the enrichment ratio; it indicates the extent of the over-representation of such functions in the reference dataset. Over-representation is highest for NCFs but clearly non-NCF RoFs are also highly over-represented. Computations are reported for functions with *k* ≤ 8 inputs.

*k*	RoF			non-NCF RoF			NCF		
	*f*_0_	*f*_1_	*E*	*f*_0_	*f*_1_	*E*	*f*_0_	*f*_1_	*E*
1	0.5	1.000	2.000	0	0	–	0.5	1.000	2.00
2	0.5	0.977	1.953	0	0	–	0.5	0.977	1.95
3	0.250	0.917	3.670	0	0	–	0.25	0.917	3.67
4	1.27 × 10^−2^	0.946	74.495	1.46 × 10^−3^	5.43 × 10^−2^	37.04	1.12 × 10^−2^	8.91 × 10^−1^	79.38
5	3.52 × 10^−6^	0.853	2.42 × 10^5^	1.04 × 10^−6^	0.083	7.99 × 10^4^	2.47 × 10^−6^	0.769	3.11 × 10^5^
6	1.91 × 10^−14^	0.776	4.06 × 10^13^	9.12 × 10^−15^	0.150	1.64 × 10^13^	9.97 × 10^−15^	0.626	6.28 × 10^13^
7	2.95 × 10^−32^	0.804	2.73 × 10^31^	1.86 × 10^−32^	0.137	7.39 × 10^30^	1.09 × 10^−32^	0.667	6.11 × 10^31^
8	2.92 × 10^−69^	0.756	2.59 × 10^68^	2.18 × 10^−69^	0.156	7.14 × 10^67^	7.41 × 10^−70^	0.600	8.10 × 10^68^

### Relative enrichment in subtypes when comparing to the ensemble of random BFs

Comparing the enrichments of the different types of biologically meaningful BFs can provide signatures of causes of enrichment. For instance, if selection operated only in favor of unateness, each subtype therein (NCF, RoF, or non-NCF RoF) would be expected to have its relative abundance (proportion within UF) be the same whether one considers the reference biological dataset or the ensemble of random BFs. In effect, the proportions of different subtypes of BFs in the 2 ensembles point to which factors drive the different enrichments. We, thus developed a way to test the null hypothesis that a subtype enrichment is solely due to the enrichment in 1 of its englobing types (see Section 6 in Supplementary Material).

Let us first consider the enrichment ratios of NCFs and RoFs within the 3 englobing types of BFs: odd bias, EFs, and UFs. From Table S9 (Supplementary Material), it is clear that, for *k* > 2, the relative enrichment ratios *E*_*R*_ (when comparing the observed to the expected under the null hypothesis) of both the NCFs and RoFs are much greater than 1, implying that the enrichment of these subtypes does not follow from the enrichment of their supersets. Thus, biological selection *solely* in favor of being odd biased, effective, or unate is not consistent with the enrichments found for the NCFs or RoFs in the reference dataset, some other factors must be at work.

Second, since NCFs are a subset of CFs, we can ask whether canalyzation is the factor driving the enrichment of NCFs. Since the relative enrichment ratios are high and the *p*-values low (see Table 2), we conclude that selection for canalyzation alone does not explain the enrichment observed for NCFs. Similarly, we can ask whether it is minimum Boolean complexity, i.e. the fact that a function is a RoF, that drives the enrichment of NCFs (a subtype of RoF). As shown in Table 2, the relative enrichment of NCF within RoF is quite modest, almost all *k* having *E*_*R*_ values in the range 1–2. Nevertheless our statistical method shows that these values are not consistent with 1 (absence of any enrichment) as indicated by the *p*-values in Table 2, so there must be some further cause of the enrichment of NCFs other than that of belonging to the RoF type.

**Table 2.** The relative enrichment ratio *E*_*R*_ of the NCFs in the CFs and RoFs. *f*_*s*,0_/*f*_0_ denotes the fractions of functions that are NCFs in the space of all CFs or RoFs and *f*_*s*,1_/*f*_1_, the equivalent fraction in the reference biological dataset. *E*_*R*_ = (*f*_*s*,1_/*f*_1_)/(*f*_*s*,0_/*f*_0_) denotes the enrichment ratio and it indicates the extent of the over-representation of such functions in the reference dataset. Computations are reported for BFs with *k* ≤ 8 inputs. The low *p*-values indicate that there is an enrichment of NCFs within the CFs and RoFs in the reference dataset when compared to that expected in the ensemble of all CFs and RoFs.

*k*	NCF in CF				NCF in RoF			
	*f*_*s*,0_/*f*_0_	*f*_*s*,1_/*f*_1_	*E*_*R*_	*p*-value	*f*_*s*,0_/*f*_0_	*f*_*s*,1_/*f*_1_	*E*_*R*_	*p*-value
1	0.5	1	2	–	1	1	1	–
2	0.571	0.977	1.709	3.49 × 10^−139^	1	1	1	–
3	0.533	0.950	1.781	2.47 × 10^−78^	1	1	1	–
4	0.209	0.962	4.595	5.32 × 10^−144^	0.885	0.943	1.066	6.86 × 10^−04^
5	8.22 × 10^−3^	0.882	1.07 × 10^2^	1.56 × 10^−233^	0.703	0.902	1.283	7.46 × 10^−09^
6	1.78 × 10^−06^	0.720	4.04 × 10^5^	0	0.522	0.807	1.546	1.58 × 10^−08^
7	7.19 × 10^−15^	0.694	9.65 × 10^13^	0	0.370	0.829	2.240	2.42 × 10^−10^
8	7.88 × 10^−33^	0.675	8.57 × 10^31^	0	0.254	0.794	3.129	5.26 × 10^−12^

## Enriched Functions in Biological Data Have Minimum Complexity

A plausible explanation for the enrichment of the RoFs and NCFs in the dataset is their *low complexity*. In terms of the first notion (Boolean complexity), the RoFs, of which NCFs are a subset, have the minimum Boolean complexity among all EFs. RoFs and NCFs have the same Boolean complexity but differ in the second measure of complexity, namely average sensitivity. This section examines more closely the properties of these 2 complexity measures. We also harness the fact that for any bias, the minimum average sensitivity is obtained for a particular geometry of the “on” vertices of the *k*-dimensional hypercube. We will show that when the bias is odd this geometry corresponds to an NCF while if it is even the function is ineffective.

### Correlation between Boolean complexity and average sensitivity

**Fig. 3:**
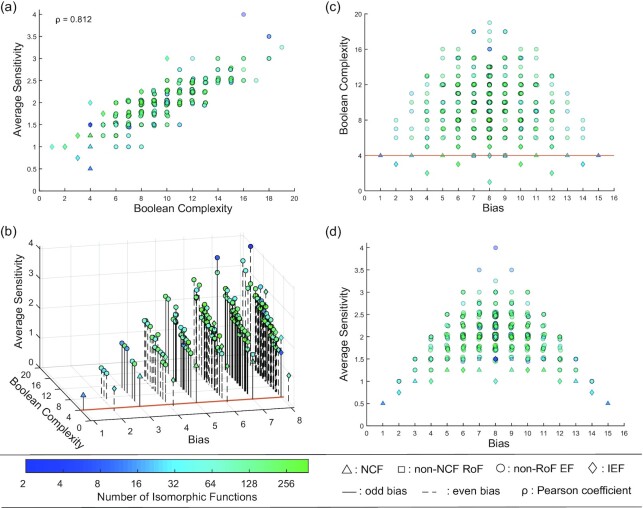
Dependence of the 2 complexity measures on the bias and associated 2D projections for all BFs with *k* = 4 inputs. In each subfigure, a point corresponds to a class of (isomorphic) BF and is assigned a shape and a color. The shape of a point (triangle, square, circle, or diamond) denotes the type of BF (NCF, non-NCF RoF, non-ROF EF, or IEF) whereas its color indicates the number of BFs contained in it’s corresponding class. The same shape and color scheme is applicable to all the plots. A slight ‘jiggle’ is added at some points to resolve overlapping representative BFs. In this plot, the type ‘non-RoF EF’ refers to the subset of EFs which are not RoFs. (a) The linear correlation between the Boolean complexity and the average sensitivity is large and positive. The Pearson correlation coefficient (ρ) between the 2 measures was calculated for all BFs with *k* = 4 and *P* ≤ 8. (b) The 3D plot adds the third dimension of bias *P* to the preceding 2D plot. The solid and dashed vertical lines or ‘needles’, as we will refer to them henceforth, show the projections of the points onto the plane of bias and Boolean complexity. These needles have been included to enhance clarity while distinguishing between the odd bias BFs and even bias BFs. The brown line drawn at the Boolean complexity 4 highlights the functions that possess the minimum Boolean complexity and are effective as well. The RoFs are the only functions which lie along this line. Since the 2 complexity measures are invariant under complementation of the BF, the bias values have been shown only up to *P* = 8. (c) Variation of the Boolean complexity with the bias. With increasing bias upto *P* = 8, the number of representative BFs increases, but so does the range of Boolean complexity of these functions. The RoFs and ineffective functions (IEFs) have the minimum Boolean complexity in any 4[*P*] set. The brown line drawn at the Boolean complexity 4 highlights the functions that possess the minimum Boolean complexity and are effective as well. (d) Variation of average sensitivity with increasing bias. Clearly, the NCFs and IEFs have the minimum average sensitivity in any 4[*P*] set. Note that both subfigures (c) and (d) are symmetric about *P* = 8 due to the complementarity property.

Let us first explore how the 2 measures of complexity compare. The average sensitivity of a BF can be computed easily using Eq. 1 while computing the Boolean complexity of a BF is more challenging but was done as described in the section on complexity measures. A bivariate analysis of these 2 measures of complexity allows us to obtain the Pearson correlation coefficient (*ρ* = 0.812) for all BFs at *k* = 4 inputs. We find that there is a strong positive linear relationship between the 2 measures (see Fig. 3(a)). Looking closely at functions in the neighborhood of the brown line (which highlights the minimum Boolean complexity of 4 for EFs) in the 3D plot Fig. 3(b), we observe that: (i) all EFs along this brown line have odd bias and are NCFs or non-NCF RoFs (see Fig. 3(b) and (c)). (ii) At bias *P* = 7, NCFs have a lower average sensitivity than the non-NCF RoFs (see Fig. 3(b) and (d)). (iii) At any even bias, the BFs having the minimum average sensitivity are IEFs of Boolean complexity strictly less than *k* (see Fig. 3(b) and (c)). These computational observations led us to the 2 conjectures listed below, which we prove in the subsequent subsections:

- When *P* is odd, NCFs have the minimum average sensitivity within their *k*[*P*] set.
- When *P* is even, the functions with minimum average sensitivity are ineffective with Boolean complexity <*k*.

### Mapping average sensitivity to the number of edges between *P* vertices of a *k*-cube

In the *k*-cube representation of a BF, each vertex corresponds to a binary string $\mathbf {x}$ that defines the BF’s input. We thus assign “0”s and “1”s to each of the associated vertices to specify the BF’s output for each input string $\mathbf {x}$. If *P* is the bias of the BF, there are *P* vertices carrying the label “1”. The total number of edges stemming from these *P* vertices is *kP*. Of these, some edges may end at one of the other *P* − 1 vertices having the value 1; we refer to the associated set of edges as *E*_11_. Similarly, we denote by *E*_01_ the remaining edges, ending at any of the 2^*k*^ − *P* other vertices having the value 0. These 2 quantities satisfy *E*_01_ + 2*E*_11_ = *kP* (44). The average sensitivity of the BF is given by 2*E*_01_/2^*k*^; clearly the problem of minimizing this quantity in the set *k*[*P*] is equivalent to maximizing *E*_11_ since *k* and *P* are fixed.

### Edge-maximizing arrangement between *P* vertices of the *k*-cube: defining “good sets”

Hart (44) solved the problem of finding an arrangement of *P* vertices on a *k*-cube that maximizes the number of edges connecting them. This problem has also been solved by other authors (45, 46), though in other contexts. We choose to use Hart’s approach due to it’s mathematical clarity and easy visualization. Hart introduces the notion of a “good set” of *P* vertices on a *k*-cube where *P* < 2^*k*^ using the following recursive definition:

- If *P* = 1, we always have a good set.
- Otherwise, find *r* such that 2^*r*^ < *P* ≤ 2^*r* + 1^. Select any (*r* + 1)-cube embedded in the *k*-cube. Then, select two *r*-cubes, which are vertex disjoint subsets of the (*r* + 1)-cube. To select the *P* vertices, include first 2^*r*^ vertices by taking one of the *r*-cubes and include the remaining *P* − 2^*r*^ vertices by imposing that they form a “good set” containing *P* − 2^*r*^ vertices on the other *r*-cube.

By expressing *P* as a sum of powers of 2, i.e. $P = \sum _{i=1}^{l} 2^{r_{i}}$, the resulting set of strictly increasing exponents {*r*_1_, *r*_2_, …, *r*_*l*_} gives the dimensions of the successive cubes to be used to define a good set. Hart (44) was able to prove that good sets maximize the number of edges connecting *P* vertices at fixed *P*.

### Good sets having an odd number of vertices correspond to NCFs

Given the *k*-cube representation of BFs in *k*[*P*], our claim is that the *P* vertices (*P* odd) with output value 1 form a “good set” iff the BF is a NCF.

*Proof*: Consider the logical expression of a NCF (Eq.   6) in a *k*[*P*] set. The *i^th^* canalyzing variable *x*_σ(*i*)_ determines which partition (of the possible *k* − (*i* − 1) partitions, *i* − 1 variables having already been fixed) of a (*k* − (*i* − 1))-cube into 2 vertex disjoint (*k* − *i*)-cubes is to be canalyzed. Furthermore, the canalyzing input value *a*_*i*_ (*x*_σ(*i*)_ = *a*_*i*_) fixes the outputs of the vertices of 1 of the 2 vertex disjoint (*k* − *i*)-cubes to the value *b*_*i*_. Repeating the procedure recursively over *i* ∈ [1, *k*] gives the arrangement of 1s and 0s for a NCF on a *k*-cube. To obtain a NCF with a certain bias *P*, the *i*’s for which *b*_*i*_ = 1 have to be chosen appropriately so that $P = \sum _{i=1}^{k} b_i 2^{k-i}$.

The above procedure of setting the output values of *P* vertices to 1s and 2^*k*^ − *P* vertices to 0s on the *k*-cube is equivalent to obtaining a good set of *P* vertices, setting their output values to 1 and then setting the output of the remaining 2^*k*^ − *P* vertices to 0. This is true because:

1. The dimensions of the cubes whose vertices are to have the output value 1 are the same in either case (i.e. the set of exponents obtained by expressing *P* as a sum of powers of 2 is unique for a given *P*).
2. When some *i*-cube is chosen to place the 1s, there is only 1 other *i*-cube, which (along with the chosen *i*-cube) constitutes 2 vertex disjoint subsets of a (*i* + 1)-cube. In both cases, this is an *i*-cube where the next set of 1s are placed.

**Fig. 4:**
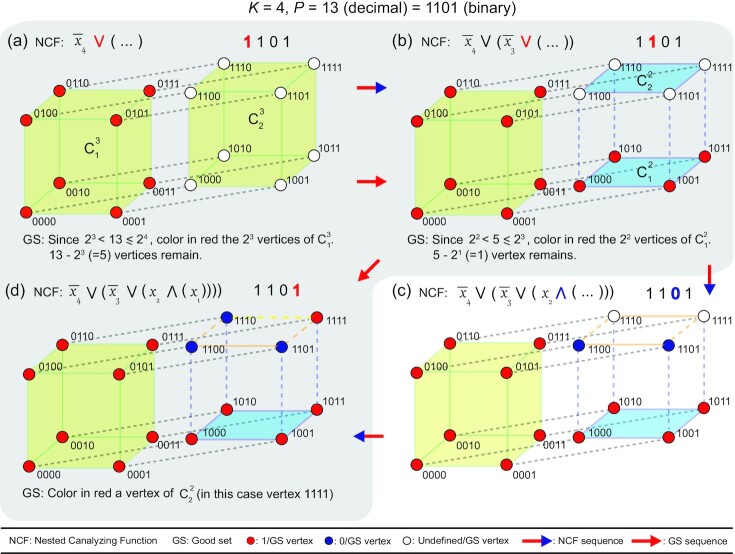
A ‘Good set’ (GS) with *P* vertices where *P* is odd on a *k*-dimensional hypercube is equivalent to a NCF in *k*[*P*]. In parts (a), (b), and (d) shaded in grey, we show the recursive construction of a GS for *P* = 13 vertices in a 4D hypercube by coloring it’s vertices red, and in parts (a), (b), (c), and (d), we show the equivalence of that GS with 13 vertices to a NCF with bias 13. The vertices of the hypercube are labeled in the order *x*_4_, *x*_3_, *x*_2_, *x*_1_, wherein *x*_*i*_ is 0 or 1. Here, $C_{1}^{j}$ and $C_{2}^{j}$ denote the 2 vertex disjoint *j*-dimensional hypercubes of the (*j* + 1)-dimensional hypercube. The ‘active’ bit in each part (a), (b), (c), and (d) is the colored bit in the binary representation of 13 in that part. (a) Since *P* = 13 lies between 2^3^ and 2^4^, 2^3^ vertices of either $C_{1}^{3}$ or $C_{2}^{3}$ (here, $C_{1}^{3}$) form part of the GS. This leaves 13 − 8 = 5 vertices to be colored red to complete the GS. This choice of 8 vertices in $C_{1}^{3}$ for the GS leads to the canalyzation of vertices labeled *x*_4_ = 0 to the output value 1. In this step, the active bit is 1 and as a result the ∨ operator follows the literal $\overline{x}_{4}$. (b) Following the same procedure as in (a) for coloring the remaining 5 vertices of the GS leads to the choice of 4 vertices in $C_{1}^{2}$. This leaves 1 vertex to be colored (which is the base case of the recursion to construct the GS). The choice of 4 vertices for the GS leads to the canalyzation of vertices with *x*_4_ = 1 and *x*_3_ = 0 to the output value 1. The active bit in this step is 1 and as a result the ∨ operator follows the literal $\overline{x}_{3}$. (c) For the corresponding NCF, the vertices with *x*_4_ = 1, *x*_3_ = 1 and *x*_2_ = 0 are canalyzed to the output value 0. The active bit in this step is 0 and as a result the ∧ operator follows the literal *x*_2_. (d) For the last step, any vertex in $C_{2}^{2}$ can be colored to complete the 13 vertices in GS, and we color here the vertex 1111. The vertex with *x*_4_ = 1, *x*_3_ = 1, *x*_2_ = 1, and *x*_1_ = 1 is canalyzed to the output value 1, and the remaining vertex is set to output value 0.

Thus the *P* vertices with output value 1 in a NCF constitute a good set and inversely any good set with *P* odd corresponds to a NCF. Given Hart’s proof, NCFs must then have the minimum average sensitivity among all BFs in *k*[*P*]. Figure 4 and Figure S6 (Supplementary Material) provide a visual illustration.

The logic of the derivation can be extended to the case where the good set has an even number of vertices: one then sees that the resulting BFs have a hierarchical structure similar to the NCFs, but with some variables ineffective (see Section 7 and Figure S7 in Supplementary Material). If all ineffective variables are ignored, one sees that a good set of even number of vertices leads to a NCF with fewer variables.

## Consequences for Network Dynamics of Biologically Meaningful BFs

**Fig. 5:**
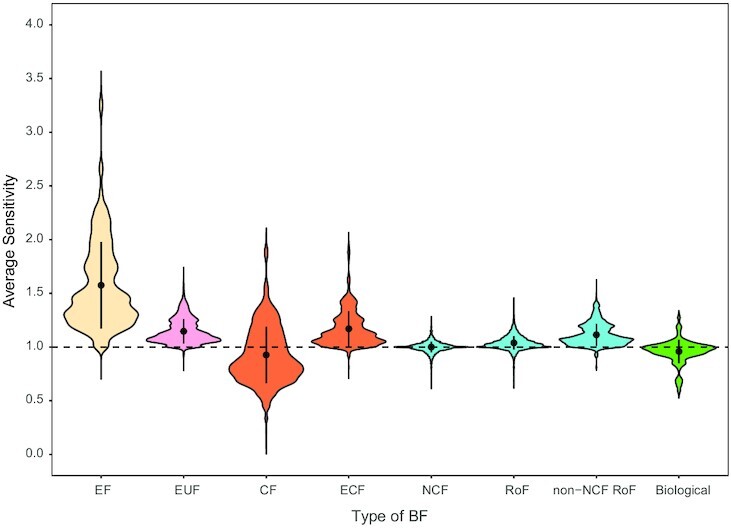
Distribution of the network average sensitivity when using the list of inputs from biological models but enforcing different types of BFs to the nodes, namely EFs, effective and unate functions (EUFs), CFs, effective and canalyzing functions (ECFs), NCFs, RoF, and non-NCF RoFs. The right-most case is the distribution when using the actual BFs in the biological models. This plot has been generated by keeping the maximum width of each of the ‘violins’ fixed.

A natural question that emerges from our results is: what are the implications of selecting these various types of BFs for the network dynamics? To answer this, we exploit the indicator defined in (22, 47) referred to as *network average sensitivity*. This quantity is the mean, over all nodes of the network, of each node’s average sensitivity. Daniels et al. (47) found that by fixing the biological network structure and selecting CFs over random BFs for all nodes, the network average sensitivity *s* of the resulting Boolean network is brought close to the critical value *s* ∼ 1. We extend this approach to consider the effects of selecting for the different biologically meaningful BFs, determining the distribution of network average sensitivities over the 88 models (see Fig. 5). We then compare these distributions to that of the biological case. By quantifying the overlaps of these different distributions, we find that all types of BFs except for the NCFs and RoFs have a substantial fraction of their distributions lying outside the $95\%$ CI of the distribution of the biological case (see Table S10, Supplementary Material). For details of these computations of network average sensitivities in 88 models and their randomized counterparts, see Section 8 in Supplementary Material. Furthermore, we see that RoFs and NCFs have rather narrow distributions that are peaked near *s* = 1 (see Fig. 5).

## Discussion and Conclusion

The first Boolean modelings of gene regulatory networks (10, 12) were based on random logic, but subsequent works introduced different types of “biologically meaningful” BFs, including effective (EFs) (38), unate (UFs) (39), canalyzing (CFs) (2), and nested canalyzing (NCFs) (19). To those types we have here added the RoFs (26) taken from the computer science literature. Furthermore, we show the relationships among these different types of BFs in: (a) the space of all $2^{2^k}$ BFs, and (b) a reference dataset of 2,687 BFs compiled from published discrete logical networks (21, 48, 49) of biological systems.

One of our main conclusions is that these biologically meaningful types of BFs represent a tiny fraction of the space of all BFs (see Fig. 1), and yet we find that they cover nearly all BFs found in our reference biological dataset (Fig. 2). Of course this dataset may reflect some biases introduced by the researchers who built the associated models but the diversity of groups involved in building these models points to the solidity of our conclusions. A cautionary note nevertheless is that the Boolean framework is an idealization of the continuous levels of the different biomolecular species. The assumption that genes are either on or off is convenient but it indeed can miss subtle effects associated with dosage dependencies. As an example, suppose gene A turns on its target gene B (respectively C) when its expression level is above the threshold Θ_*B*_ (respectively Θ_*C*_). If Θ_*B*_ < Θ_*C*_, the regime where A turns on B but not C cannot be handled within the Boolean framework. In view of such caveats that are not specific to the present work, our results should be anchored in their context, namely a coarse-grained conceptual framework approximating reality.

Another major conclusion we reach is that RoFs and their subset NCFs are specifically and strongly enriched in the reference dataset. We remark that while the relative abundance of CFs and NCFs in biological networks has been previously reported in several publications (2, 19, 20, 43, 47, 50, 51), our work provides a systematic study of 7 different types of BFs in a large curated reference biological dataset. In fact, previous studies neither carried out statistical tests nor assessed the relative enrichments in subtypes, e.g. NCFs within CFs or RoFs, and in this respect, our study is able to shed light on possible factors driving enrichment. The specific enrichment of RoFs and NCFs can be tied to their minimizing 2 measures of complexity namely, Boolean complexity (24, 30) and average sensitivity (22, 25). RoFs turn out to be the set of BFs minimizing Boolean complexity. Furthermore, extending previous studies realizing that NCFs have low average sensitivity (23, 42, 52), we show that in fact NCFs achieve the *theoretical minimum* of this complexity measure in their *k*[*P*] set, a result that was also reported in a recent preprint (53).

In the reference dataset, we found occurrences of ineffective BFs even though the corresponding models had been curated by their authors. Most likely such cases are modeling errors. A possible way to handle an ineffective BF in such a biological context is by considering the truncated BF without its ineffective inputs. We have confirmed that all our conclusions remain unchanged by repeating the analysis starting with a modified reference dataset, wherein every ineffective BF is replaced by its corresponding truncated effective BF (see Section 9, Tables S11–S17, Figures S8 and S9 in Supplementary Material).

Buchler et al. (54) provide a biophysical model of how regulatory logic schemes could be realized at any node in a gene regulatory network. They recognize via their model that implementing the XOR and XNOR logic is more complex than implementing AND and OR logic. This is in concordance with what our complexity measures furnish: the Boolean complexity and average sensitivity of XOR and XNOR functions are both greater than that of AND and OR functions. Moreover, the XOR and XNOR functions have no representation among the 687 2-input BFs in the reference biological dataset. Altogether, these observations support the use of certain representations of BFs in the biological scenario, wherein variables are connected by either conjunction or disjunction operators, in contrast to other representations wherein say the variables are connected by the XOR operator.

The framework we use both supports and formalizes Kauffman’s (2) qualitative view in which “simplicity” should be a driver of the regulatory logic in biological systems. Kauffman argued that CFs were simpler than random functions, and therefore, should be expected to arise quite frequently in biological systems (2, 50). Our use of an extensive curated dataset generated from published Boolean models of biological networks enabled us to compare different notions of simplicity, and thereby confront Kauffman’s view to real data in a well defined quantitative framework. By identifying “simplicity” with minimum complexity defined in terms of either Boolean complexity or sensitivity, NCFs are the simplest of all BFs. We can, thus justify the much stronger preponderance of the NCF type in comparison to the CF type conjectured by Kauffman.

We also note that sensitivity of BFs is directly related to their robustness to noise (6). With that correspondence, we can conclude that NCFs for a given number of inputs *k* and given bias *P* have the *theoretically maximum robustness* to noise in the inputs. It is a posteriori natural to expect that average sensitivity as a measure of both complexity and robustness will be particularly relevant to Boolean models of gene regulatory networks.

As a caveat or at least as a subtlety to our minimum complexity conclusion, it is appropriate to stress that NCFs minimize average sensitivity within their *k*[*P*] set, that is at fixed bias *P*. Since lowering bias *P* could lead to a lower average sensitivity, one may ask why there are cases where *P* is large in the biological reference dataset. We speculate that the answer has to do with what function the biological network implements. To use a parallel from electronics, it is possible for a circuit to implement a function by using many simple components or by using fewer but more complex components. The relative advantage of each strategy depends on component “costs”. In the biological context one may expect that having higher values of *P* allows one to use fewer genes, thereby reducing protein and cellular machinery costs. Tackling this question in a quantitative framework will be very challenging and it is definitely beyond the scope of this paper.

Lastly, our methods and results have implications for the problem of model selection within the Boolean framework (55, 56). By model selection we mean the process of selecting Boolean models from the ensemble of Boolean models which satisfy given constraints such as having specified steady states. During model selection, the preferential use of NCFs or RoFs could serve as a relevant criterion to constrain network reconstruction (55, 57).

## Supplementary Material

pgac017_Supplemental_File

## Data Availability

All datasets and programs needed to reproduce the results of this study are available from the associated GitHub repository: https://github.com/asamallab/MCBF.

## Preprints

A preprint of this article has previously appeared at: https://doi.org/10.1101/2021.09.20.461164.
