# Supplementary material for: Minimum complexity drives regulatory logic in Boolean models of living systems
Source: PNAS Nexus. 2022 Apr 15;1(1):pgac017. doi: 10.1093/pnasnexus/pgac017 (PMC9802451; doi:10.1093/pnasnexus/pgac017)
Supplement: pgac017_Supplemental_File [file pgac017_supplemental_file.pdf]

SUPPLEMENTARY MATERIAL

FOR

Minimum complexity drives regulatory logic in Boolean models of living systems

Ajay Subbaroyan,[a,b] Olivier C. Martin[c,d, *] and Areejit Samal[a,b, *]

[a]The Institute of Mathematical Sciences (IMSc), Chennai, 600113, India, [b]Homi Bhabha National Institute (HBNI), Mumbai, 400094, India, [c]Université Paris-Saclay, CNRS, INRAE, Univ Evry, Institute of Plant Sciences Paris-Saclay (IPS2), 91405, Orsay, France and [d]Université de Paris, CNRS, INRAE, Institute of Plant Sciences Paris-Saclay (IPS2), 91405, Orsay, France

[*]To whom correspondence should be addressed: olivier.c.martin@inrae.fr, asamal@imsc.res.in

1. Representation of Boolean functions

Let $f = f(x_1, x_2, \dots, x_k)$ be a BF of k input variables where each variable $x_i \in \{0, 1\}$. A BF maps 2^k different possibilities for the k input variables to output values 0 or 1, i.e., $f : \{0, 1\}^k \mapsto \{0, 1\}$.

Truth table and associated ordered binary vector

A BF f with k inputs can be captured in the form of a truth table with 2^k rows, each corresponding to a possible state of the set of k input variables. In our convention the last entry of each row gives the output value for the corresponding state of the input variables (Fig. S1(a)). Thus, a BF can be stored as a binary vector of size 2^k, where each element of the vector corresponds to the output value of the corresponding row of the truth table (Fig. S1(b)). A BF can also be encoded as the integer which is the decimal equivalent of the binary vector of size 2^k. Since the output value for each of the 2^k different states of the k input variables can take either of two values, 0 or 1, the number of possible BFs f with k inputs is 2^{2^k}. Thus, the number of possible BFs f blows up quickly with increasing k [1], e.g., there are over 10^9 BFs with $k = 5$ inputs (see Table S1 and Fig. S2).

Boolean Expression

Alternatively, a BF f can instead be represented as an algebraic expression (Fig. S1(c)) constructed with the k input variables which are combined via the logical operators AND ($\cdot$ or $\wedge$), OR ($+$ or $\vee$) and NOT ($'$ or $\bar{\ }$). For example, the AND function and OR function of 2 input variables, x_1 and x_2, are given by the Boolean expressions, $x_1 \cdot x_2$ and $x_1 + x_2$, respectively. Note that the "+" symbol does not correspond to working modulo 2, instead (1+1) has the value "1", not "0". In this work, the term *literal* refers to a Boolean variable (e.g., x_1) or the complement of a Boolean variable (e.g., $\overline{x}_1$). Throughout this work, *Boolean function* and *Boolean variables* refer to a *logical update rule* and to *inputs*, respectively, of nodes in a model.

Colored Boolean Hypercube

A visually illustrative representation of a BF is obtained by coloring a Boolean hypercube. A k-dimensional hypercube (k-cube) is composed of vertices and edges where each vertex is labelled by a string of k bits, and is connected to vertices with labels that differ from its label in exactly one bit. Two vertices connected by an edge are called "neighbors". A k-cube thus has 2^k vertices, with each vertex having k neighbors. The total number of edges in a k-cube is $(k \times 2^k)/2$ (division by 2 removes the double counting edges). A BF may thus be represented by a k-cube in which each vertex is labeled by the input combination $x_k x_{k-1} x_{k-2} \dots x_2 x_1$ ($x_i \in \{0, 1\}$) and is colored with an output bit (0 or 1) (Fig. S1(d)).

2. Combining two independent Boolean functions

Consider two *independent* BFs f_1 and f_2 with k_1 and k_2 inputs and bias P_1 and P_2, respectively. Here, the two BFs are independent in the sense that they have no input variables in common. The truth tables for the BFs f_1 and f_2 have 2^{k_1} and 2^{k_2} rows, respectively. A simple way to combine the two BFs is via the AND or OR logical operators. We use the notation $f = f_1 \odot f_2$ where $\odot$ is either the AND ($\wedge$) or OR ($\vee$) operator. The procedure to generate f with $2^{k_1+k_2}$ rows in its truth table, by combining f_1 and f_2, can be expressed compactly as follows:

© The Author . Published by Oxford University Press. All rights reserved. For permissions, please e-mail: journals.permissions@oup.com

Algorithm 1 Algorithm to combine two independent BFs f_1 and f_2

1: $f[r] = 0, \;\; r \in [1, 2^{k_1+k_2}]$
2: $r \leftarrow 1$
3: **for** $i \leftarrow 1$ to 2^{k_1} **do**
4:     **for** $j \leftarrow 1$ to 2^{k_2} **do**
5:         $f[r] = f_1[i] \odot f_2[j]$
6:         $r \leftarrow r + 1$
7:     **end for**
8: **end for**

In Line 1 of the above algorithm, we initialize a vector f with $2^{k_1+k_2}$ elements to store the output values in each row of the truth table for $f = f_1 \odot f_2$. In Line 5 of the algorithm, the output value for the ith row of f_1 is combined with that of the jth row of f_2 to give the output value for the rth row of f. For example, if BFs f_1 and f_2 with 1 input and 2 inputs, respectively, have output vectors $(1, 0)$ and $(1, 1, 1, 0)$, respectively, then the output vector for the combined BF $f = f_1 \wedge f_2$ with 3 inputs is $(1, 1, 1, 0, 0, 0, 0, 0)$.

Property 2.1. Let f_1 and f_2 have bias P_1 and P_2. Then $f_1 \wedge f_2$ (hereafter denoted as f_{AND}) has bias equal to $P_1 P_2$.
Proof: For every occurrence of 0 in the output vector of f_1, the output vector of f_{AND} will also be 0. For every occurrence of 1 in the output vector of f_1, there will be P_2 occurrences of 1 in the output vector of f_{AND}. Thus, for P_1 occurrences of 1 in the output vector of f_1, there will be $P_1 P_2$ occurrences of 1 in the output vector of f_{AND}.

Property 2.2. Let us denote $f_1 \vee f_2$ by f_{OR}, then f_{OR} has bias equal to $2^{k_1} P_2 + 2^{k_2} P_1 - P_1 P_2$.
Proof: For every occurrence of 0 in the output vector of f_1, there will be P_2 occurrences of 1 in the output vector of f_{OR}. Thus, the contribution to the 1's in the output vector of f_{OR} from 0's in the output vector of f_1 is $(2^{k_1} - P_1)P_2$. For every occurrence of 1 in the output vector of f_1, there will be 2^{k_2} occurrences of 1's in the output vector of f_{OR}. Thus, the contribution to the 1's in the output vector of f_{OR} from 1's in the output vector of f_1 is $2^{k_2} P_1$. In total, the number of 1's in the output vector of f_{OR} is equal to $(2^{k_1} - P_1)P_2 + 2^{k_2} P_1 = 2^{k_1} P_2 + 2^{k_2} P_1 - P_1 P_2$.

Property 2.3. Given the bias parities (even or odd) of f_1 and f_2, the two previous results show that both f_{AND} and f_{OR} have *odd* parity (i.e., their bias is odd), if and only if P_1 and P_2 are both odd.

3. Properties of biologically meaningful types of Boolean functions

Here, we present proofs for various properties of EFs, UFs, NCFs and RoFs.

Effective functions

Property 3.1. The bias P of a BF with m ineffective inputs is a multiple of 2^m.
Proof: Consider the truth table of a BF f and assume the input variable x_i is ineffective. Then each line with $x_i = 0$ can be uniquely paired with the corresponding line having $x_i = 1$ where all other variables are unchanged. Since the output is the same in both of these lines, one has either no 1s or two 1s in the output. Summing over all of the truth table (lines coming in pairs) will thus lead to an even number of 1s. The result for m ineffective inputs is then obtained by recurrence.
Corollary: It immediately follows that a BF with an odd bias P is effective.

Property 3.2. EFs with k inputs have Boolean complexity $\geq k$.
Proof: For a BF f to be effective, the k different input variables must appear at least once in the minimal expression or formula for the BF. This implies that the number of literals in the minimal expression or Boolean complexity is $\geq k$.

Unate functions

Property 3.3. A UF can be represented by an expression in disjunctive normal form (DNF) in which all occurrences of any specific input variable (more precisely, literal) are either negated (i.e., negative input) or non-negated (i.e., positive input) [2, 3].

Property 3.4. If u_1 and u_2 are UFs with k_1 and k_2 independent input variables, respectively, then the combined BF $u = u_1 \odot u_2$ is also unate.
Proof: Consider two unate functions u_1 and u_2. For convenience, let us denote their DNF expressions by the same symbols u_1 and u_2. Since each input variable in u_1 and u_2 is either a positive (x_i) or a negative $(\overline{x}_i)$ literal (see Property 3.3), the combined expression $u = u_1 \odot u_2$ composed of $(k_1 + k_2)$ distinct input variables (due to independence) will also have each variable occur as only its positive or negative literal. This implies that the combined function u is a UF. As an example, consider the UFs $u_1 = (x_1 + \overline{x}_2)$ and $u_2 = (x_3 \overline{x}_4 + x_5)$. The combined BF $u = u_1 \odot u_2$ under the AND operation is simply $u = (x_1 + \overline{x}_2)(x_3 \overline{x}_4 + x_5)$. Since each literal appears in u only in its positive or its negative form in the expression for u, the combined BF is UF.

Property 3.5. If an input i of a UF u acts as both an activator and an inhibitor, then input i is ineffective.
Proof: An input i acts as both an activator and an inhibitor if and only if the input i satisfies the equality condition in Eqs. 3 and 4 in the main text, respectively, for all input vectors $\mathbf{x}$. However, this is precisely equivalent to the condition for an input i to be ineffective.

Canalyzing and Nested Canalyzing functions

Canalyzing functions must have at least one canalyzing input. CFs have been grouped based on the number of canalyzing inputs [4]. The distinct number of inputs that satisfy the canalyzing property is referred to as the function's *canalyzing depth.* A k-input CF has a canalyzing depth ranging from 1 to k. Note that a k-input NCF has a canalyzing depth of k. A commonly used definition of the NCFs is provided below. A BF f with k inputs is *nested canalyzing* with respect to a permutation σ on its inputs $\{1, 2, \ldots, k\}$ if:

$$f(\mathbf{x}) = \begin{cases} b_1 & \text{if } x_{\sigma(1)} = a_1, \\ b_2 & \text{if } x_{\sigma(1)} \neq a_1, x_{\sigma(2)} = a_2, \\ b_3 & \text{if } x_{\sigma(1)} \neq a_1, x_{\sigma(2)} \neq a_2, x_{\sigma(3)} = a_3, \\ \vdots & \\ b_k & \text{if } x_{\sigma(1)} \neq a_1, x_{\sigma(2)} \neq a_2, \ldots, x_{\sigma(k)} = a_k, \\ \overline{b}_k & \text{if } x_{\sigma(1)} \neq a_1, x_{\sigma(2)} \neq a_2, \ldots, x_{\sigma(k)} = \overline{a}_k. \end{cases} \tag{1}$$

In the above equation, $a_1, a_2, \ldots, a_k$ are the canalyzing input values and $b_1, b_2, \ldots, b_k$ are the canalyzed output values for inputs $\sigma(1), \sigma(2), \ldots, \sigma(k)$ in the permutation σ of the k inputs. Here, $\overline{a}_k$ and $\overline{b}_k$ are the complements of the Boolean values a_k and b_k, respectively. The Boolean expression for the NCFs is given by the equation:

$$f(\mathbf{x}) = X_{\sigma(1)} \odot (X_{\sigma(2)} \odot (X_{\sigma(3)} \odot \ldots (X_{\sigma(k-1)} \odot X_{\sigma(k)}))) \tag{2}$$

where σ is a permutation on the inputs $\{1, 2, \ldots, k\}$, $X_{\sigma(i)} \in \{x_{\sigma(i)}, \overline{x}_{\sigma(i)}\}$ and $\odot \in \{\wedge, \vee\}$. Here $\wedge$ and $\vee$ are the AND and OR operators respectively. We remark that Szallasi and Liang [5] had called these functions "hierarchically canalyzing" and subsequently Kauffman [6] called them "nested canalyzing".

Property 3.6. NCFs have odd bias.
Proof: Consider the base case of a NCF with 1 input with the representative expression $NCF(1) = x_1$. Clearly, the Boolean expressions $f = x_1$ and $f = \overline{x_1}$ refer to the BFs with output vector $(0, 1)$ or $(1, 0)$, both of which have odd bias. Next, let us hypothesize that all NCFs with k inputs, i.e., $NCF(k)$, have odd bias P. We can then proceed by induction. A NCF with $k+1$ inputs is given by $NCF(k+1) = x_{k+1} \odot NCF(k)$ by definition (Eq. 2). Since the two independent BFs x_{k+1} and $NCF(k)$ have odd bias, using Property 2.3, the combined BF $NCF(k+1)$ will also have an odd bias. Note that a different proof for this property of NCFs was provided by Nikolajewa *et al.* [7].

Property 3.7. NCFs are EFs.
Proof: Since BFs with odd bias are EFs, using Properties 3.1 and 3.6, NCFs are also EFs.

Property 3.8. NCFs are UFs [3].
Proof: Following Aracena [3], since each variable or literal in the expression for a NCF (Eq. 2) appears exactly once, it follows that each variable is fixed to either its positive or negative form in the function's canonical NCF form. Thus, NCFs are UFs using Property 3.3.

Property 3.9. NCFs with k inputs have Boolean complexity equal to k.
Proof: In Eq. 2, each variable or literal in the expression for an NCF appears exactly once, thus the Boolean complexity of a NCF is equal to k. Thus, NCFs have the *minimum* Boolean complexity among EFs with given number of inputs.

Read-once functions

RoFs are also known as fanout-free functions in the computer science literature [8]. Mathematically, a k-input BF f is a RoF if, after stripping of all parentheses, there exists a permutation σ on $\{1, 2, \ldots, k\}$ such that

$$f(\mathbf{x}) = X_{\sigma(1)} \odot X_{\sigma(2)} \odot X_{\sigma(3)} \ldots \odot X_{\sigma(k)} \tag{3}$$

where $X_{\sigma(i)} \in \{x_{\sigma(i)}, \overline{x}_{\sigma(i)}\}$ and $\odot \in \{\wedge, \vee\}$. There are no restrictions on the placement of the parentheses between the variables in the above equation. Here $\wedge$ and $\vee$ are the AND and OR operators respectively.

Property 3.10. Generation of representative RoFs.
The following is a recursive scheme to generate all RoFs with k inputs, i.e., $RoF(k)$, starting from RoFs with 1 input ($RoF(1)$). To do so, one can use the fact that the parentheses in the logical expression of a function in $RoF(k)$ define two sub-parts separated

by an AND or an OR operator. Such a decomposition splits the k variables into two sets, and thus, any function in $RoF(k)$ can be decomposed into at least one of the following types:

$$RoF(k-1) \odot RoF(1)$$
$$RoF(k-2) \odot RoF(2)$$
$$RoF(k-3) \odot RoF(3)$$
$$\vdots$$
$$RoF(k-(k/2)) \odot RoF(k-(k/2))$$
$$[\text{for } k \text{ even}]$$
$$\text{or}$$
$$RoF(k-((k-1)/2)) \odot RoF(k-((k+1)/2))$$
$$[\text{for } k \text{ odd}]$$

where $\odot$ corresponds to the AND ($\wedge$) or OR ($\vee$) operator. Such a decomposition allows one to enumerate all elements of $RoF(k)$ recursively.

The above algorithm does not only return the representative RoFs, sometimes it will return permutations thereof. To retain only the representative RoFs, we iteratively walk through the produced list and keep an element only if it is not equivalent to a previous element under permutation of the variables.

Property 3.11. RoFs have odd bias.
Proof: Consider the base case of a RoF with 1 input. Clearly, the BFs in $RoF(1)$ have output vector $(0,1)$ or $(1,0)$, both of which have odd bias. Next, let us hypothesize that BFs in $RoF(j)\ \forall\ j\ \in \{1,k\}$ have odd bias. We now refer to Property 2.3 in Section 2 whereby the combination of two BFs with odd bias results in a BF with odd bias. Next by induction, the RoF with $k+1$ inputs is given by $RoF(k+1) = RoF((k+1)-j) \odot RoF(j)$ for some $j \in [1,(k+1)/2]$ for odd k, or $j \in [1,k/2]$ for even k (see Property 3.10). Since the two functions $RoF((k+1)-j)$ and $RoF(j)$ have odd bias, using Property 2.3, the function $RoF(k+1)$ will also have odd bias.

Property 3.12. RoFs are EFs.
Proof: From Property 3.11, RoFs have odd bias. From Property 3.1, BFs with odd bias are EFs. Thus, RoFs are EFs.

Property 3.13. RoFs are UFs.
Proof: Since each variable or literal in the expression for a RoF (Eq. 3) appears exactly once, it follows that each variable is fixed to either its positive or negative form in the RoF logical expression. Thus, RoFs are UFs according to Property 3.3.

Property 3.14. RoFs with k inputs have the minimum Boolean complexity k among all the EFs.
Proof: Since RoFs are constructed such that each variable or literal in the expression for a RoF (Eq. 3) appears exactly once, the Boolean complexity of a RoF is equal to k. Further, using property 3.2, k is the minimum value in EF, hence RoFs have the minimum Boolean complexity among all EFs. In sum, RoFs correspond exactly to the set of EFs with *minimum* Boolean complexity.

Property 3.15. For any k, NCFs are a subset of RoFs.
Proof: Comparing the expression for NCFs (Eq. 2) with the expression for RoFs (Eq. 3), it is evident that NCFs form a subset of RoFs. Simply stated, all NCFs are RoFs but all RoFs need not be NCFs. Henceforth, we refer to the subset of the RoFs which are not NCFs as the 'non-NCF RoFs'. To the best of our knowledge, RoFs (excluding NCFs) have not been considered in the biological literature.

Property 3.16. RoFs with bias P equal to 1, 3 and 5 are NCFs.
Proof: First consider the case where the bias P is 1. The DNF of a BF with k inputs and bias P equal to 1 has just one term, the conjunction of k literals, and thus, the function is a NCF (see Eq. 2).

Next, we show that it is impossible to have a RoF with $k > 2$ (respectively, $k > 3$) and bias $P = 3$ (respectively $P = 5$) by combining RoFs with the OR operator. Let P_{OR} be the bias of $RoF_{\text{OR}}(k) = RoF(k_1) \vee RoF(k_2)$, where $k = k_1 + k_2$. Further, let P_1 and P_2 be the biases of $RoF(k_1)$ and $RoF(k_2)$, respectively. From Property 2.2, we have $P_{OR} = 2^{k_1}P_2 + 2^{k_2}P_1 - P_1P_2$, which is a positive monotonic function of P_1 and P_2 (for a fixed k_1 and k_2). The minimum value of P_{OR} is thus obtained at $P_1 = 1, P_2 = 1$. Thus, $min(P_{\text{OR}}) = 2^{k_1} + 2^{k_2} - 1$. If k is greater than 2 (or 3), it can be easily confirmed that $min(P_{\text{OR}}) > 3$ (respectively, $min(P_{\text{OR}}) > 5$). Thus, the bias of RoF_{OR} for $k > 2$ (respectively, $k > 3$) cannot be 3 (respectively, 5).

Thus, it follows that a RoF with $k > 2$ (respectively, $k > 3$) and bias 3 (respectively, 5) can be generated only by combining RoFs with the AND operator. Let P_{AND} be the bias of $RoF_{\text{AND}}(k) = RoF(k_1) \wedge RoF(k_2)$, where $k = k_1 + k_2$. From Property 2.1, $P_{AND} = P_1P_2$. Since 3 (respectively, 5) is prime, a $RoF_{\text{AND}}(k)$ with bias 3 (respectively, 5) can be generated only by combining two RoFs, $RoF(k_1)$ and $RoF(k_2)$, with biases 1 and 3 (respectively, 1 and 5). Let $RoF(k_2)$ have bias 3 (respectively, 5). By

decomposition, $RoF(k_2)$ would in turn have to be generated by combining two RoFs, $RoF(k_{2,1})$ and $RoF(k_{2,2})$, with biases 1 and 3 (respectively, 1 and 5), and so on. Proceeding in this manner, we will be left with a *nested* RoF, with exactly one term having bias 3 (respectively, 5), and all other RoFs in the *nested* expression having bias 1. In other words, for bias 3 *nested* RoF would be of the form: $x_1 \wedge x_2 \wedge x_3 \wedge \ldots \wedge x_{k-2} \wedge (x_{k-1} \vee x_k)$, and for bias 5 would be of the form: $x_1 \wedge x_2 \wedge x_3 \wedge \ldots \wedge x_{k-3} \wedge (x_{k-2} \vee (x_{k-1} \wedge x_k))$, both of which are NCFs (see Eq. 2).

Note that for $k = 1$, there are no BFs with bias P equal to 3. To complete the proof, consider the cases $k = 2$ and $k = 3$. For $k = 2$, RoFs with bias $P = 1$ are NCFs, hence its complement (with bias $P = 3$), is also a NCF. For $k = 3$, RoFs with bias $P = 5$ are NCFs since it is the complement of bias $P = 3$ which we showed to be NCFs for all values of k.

In Fig. 3(d), at k=4, we find that when a NCF and a non-NCF RoF belong to the same $k[P]$ set, then the NCF has a lower average sensitivity compared to the non-NCF RoF. Further, we have shown that NCFs have the *minimum* average sensitivity in any $k[P]$ set with odd bias P. We were curious as to whether two representative non-NCF RoFs within a $k[P]$ set could have the same average sensitivity, and we find this is indeed true via exhaustive computational enumeration of RoFs with $k \leq 10$. We observe that such a case of two representative non-NCF RoFs first occur when $k = 7$ at the bias $P = 25$. In Section 4, we describe our program to check if a user-specified BF is a RoF.

For the sake of compactness, we represent RoFs which are equivalent up to isomorphisms (i.e., permutations of indices and complementation of input variables) via a single representative BF or expression. Furthermore, we classify RoFs into $k[P]$ sets based on the number of inputs k and bias P [9]. In other words, we capture the complete set of RoFs in different $k[P]$ sets via representative RoFs wherein each representative RoF captures all RoFs that are equivalent up to isomorphisms. For example, among BFs with $k = 4$ inputs, there are 10 representative RoFs up to isomorphisms which are:

RoF	Expression	$k[P]$ set
f_1	$x_1x_2x_3x_4$	4[1]
f_2	$x_1x_2(x_3 + x_4)$	4[3]
f_3	$x_1(x_2 + x_3x_4)$	4[5]
f_4	$x_1(x_2 + x_3 + x_4)$	4[7]
f_5	$x_1x_2 + x_3x_4$	4[7]
f_6	$x_1 + x_2x_3x_4$	4[9]
f_7	$(x_1 + x_2)(x_3 + x_4)$	4[9]
f_8	$x_1 + x_2(x_3 + x_4)$	4[11]
f_9	$x_1 + x_2 + x_3x_4$	4[13]
f_{10}	$x_1 + x_2 + x_3 + x_4$	4[15]

Among the above-mentioned 10 RoFs with $k = 4$, f_1, f_2, f_3, f_4, f_6, f_8, f_9 and f_{10} are also NCFs.

4. RoF checker

To check whether a BF is a RoF, we make use of the various properties of RoFs. To begin with, we generate a representative RoF for each equivalence class, going up to $k = 10$ inputs using the Property 3.10. We store the truth table, bias and the average sensitivity of each representative RoF in computer memory so that it can be used as a lookup table. Next, we implement the procedure shown in the flowchart (see Fig. S5). This program takes as input a BF via its truth table representation; the bias of the BF is determined. The program then proceeds by performing successive tests, from quite simple to more complex, as follows. If the bias is even then the BF is not a RoF (see Property 3.11). Since NCFs are a subset of RoFs, we check whether the BF is a NCF as that is relatively simple computationally (just successively determine the canalyzing input variables). If the function is not a NCF, we check whether the input BF is a UF since RoFs are unate. If the BF is unate, we calculate average sensitivity. Then we use the lookup table to extract all of the representative RoFs having that bias and average sensitivity. Recall that all elements in an equivalence class have the same bias and average sensitivity. In case no representative RoF matches, then the BF is not a RoF. Assuming that there is at least one representative RoF extracted, the program then loops over that list. For each such RoF we generate all RoFs belonging to that same equivalence class (just loop over all isomorphisms), and for each such function the program directly checks whether its truth table is the same as that of the input BF. If an equality is found, the input BF is a RoF and we are done. If all the representative RoFs are tested without any success, then the input BF is not a RoF. The catalog of RoFs along with the python code to check for RoFs is available via the GitHub repository: `https://github.com/asamallab/MCBF`.

5. Biological dataset compiling Boolean functions from reconstructed discrete models of living systems

To assess the abundance of different types of biologically meaningful BFs in reconstructed discrete models of living systems, we first compiled a large dataset of 88 models that have been published to date. These 88 models were either downloaded from databases such as Cell Collective [10] (`https://cellcollective.org/`), GINSIM [11] (`http://ginsim.org/`) or BioModels [12] (`http://www.ebi.ac.uk/biomodels/`), or directly obtained from the corresponding published article. Notably, most of these 88 models were downloaded from the Cell collective database [10]. Further, this compilation of 88 models spans the overwhelming majority of Boolean models of biological systems reconstructed and published to date. The majority of these models pertains to mammalian systems and a much smaller fraction pertains to plant systems. The mammalian models include networks for signaling pathways [13, 14, 15], differentiation [16, 17] and various cancers [18, 19, 20]. Among the plant models, this compilation includes cases from

flower organ specification [17], root stem cells [21], and guard cell signalling [22]. Overall, the 88 discrete models in this compilation capture a very diverse collection of biological processes throughout multiple kingdoms of life.

This study is focused only on properties of BFs assigned to different nodes in reconstructed models of biological networks. Some of those networks included nodes taking more than two discrete states; in our compilation, we included only BFs assigned to nodes with binary states which further also had inputs only from other nodes with binary states. While compiling the BFs from these 88 models, we have also gathered the information on the *signs* of interactions between regulators (input nodes) and target gene (output node). Such information is typically obtained from associated experimental literature.

Across the 88 models in this compilation, the number of nodes in a model varies between 4 and 128. From these 88 models, we have compiled 2687 BFs pertaining to 2687 nodes that have number of inputs $k \geq 1$ (Fig. 2(b)). The BFs assigned to each node in these 88 models are the result of many authors manually identifying appropriate input-output relations during network reconstruction. In other words, the 2687 BFs in the reference biological dataset were chosen during model reconstruction process such as to capture the known regulatory information. This reference dataset of 2687 BFs is available via the GitHub repository: `https://github.com/asamallab/MCBF`.

6. Statistical Tests

Enrichments and relative enrichments

Consider a given type of BF (say unate with k inputs) which we refer to as T. Denote by f_0 the fraction of functions that are of type T in the random ensemble and by f_1 the corresponding fraction in our reference biological dataset. The enrichment ratio E is simply f_1/f_0. If $E > 1$, then T is enriched while if $E < 1$ T is depleted. If $E = 1$, there is neither enrichment nor depletion.

In our study we are also interested in relative enrichments to probe for possible causes of enrichments. For that we consider a type T and one of its sub-types, say T_s. For instance in our comparison of the two measures of complexity we examined the case where T=RoF and T_s=NCF. In direct analogy with what was done for enrichments, we define the relative enrichment $E_R = (f_{s,1}/f_1)/(f_{s,0}/f_0)$ where the subscript s refers to type T_s. If biological enrichment is driven solely by the property of being in T, then the relative enrichment is expected to be close to 1. As a consequence, if E_R is large, then there must be other factors than “belonging to T” driving this relative enrichment.

Associated p-values

We developed a first statistical test to determine whether an observed enrichment E was statistically significant. The underlying statistical distribution of the random variable E is obtained by formalizing an underlying hypothesis referred to as H_0. Here H_0 corresponds to hypothesizing that the functions in the reference biological dataset are drawn from the random ensemble where all 2^{2^k} BFs with k inputs are equiprobable. The (right-sided) p-value is then just the probability that such a drawing leads to a value of E as large as the one actually observed. This probability is computed as follows. The fraction f_0 is first determined (see Table S2). Then we consider drawing M BFs from the random ensemble and count the number m of these functions that belong to type T (M is the number of BFs in the reference biological dataset). The probability of having a given value m is given by the binomial distribution: $\binom{M}{m} f_0^m (1-f_0)^{M-m}$. The desired p-value is then just the sum of all such probabilities under the condition that m is larger or equal to Mf_1. This sum is computed numerically.

The second type of test we perform concerns the statistical significance of a relative enrichment E_R deviating from 1. Again we formalize this by introducing an H_0 hypothesis. Using the notation of the previous subsection, H_0 corresponds to assuming that although there is a selection for T (as evident from a large value of E), the elements that are drawn within T have a uniform probability, that is members of T_s are not more probable than the other elements of T. Consider then drawing a sample of size M under H_0. If it leads to M_T elements in T as in the reference biological dataset, the distribution of the number of elements in T_s is known. Specifically, the probability to have m elements in T_s is $\binom{M_T}{m} f_0^m (1-f_0)^{M_T-m}$ where now f_0 is the ratio of sizes of T_s and T (see Table S2). The desired p-value is then just the sum of all such probabilities under the condition that m is larger or equal to the number of T_s elements in the reference biological dataset. Again, this sum is computed numerically.

The number of functions belonging to a particular type of BF was obtained from both computation and theory. The number of CFs for $k = 6, 7, 8$ and NCFs for $k = 7, 8$ were obtained from [23] and [24]. In certain cases (EFs and UFs having $6, 7$ or 8 inputs), it was computationally unfeasible to obtain the exact number of functions in these types and there was no data in the literature as well, hence we used sampling to estimate the probability of a BF to belong to these types, for the specified number of inputs.

7. A“good set” having an even vertices has Boolean complexity strictly less than k

Claim: If an even number of vertices P having the output value 1 in the hypercube representation of a BF (in a $k[P]$ set) forms a “good set”, then its Boolean complexity is strictly less than k.
Proof: The arrangement of P 1s and $2^k - P$ 0s on the k-cube in the case where P is even is almost the same as in a NCF, with the exception that the vertices of the last 1-cube (composed of 2 vertex disjoint sets of 0-cubes) will have the same output values b_k. By direct computation, we have $P = \sum_{i=1}^{k} b_i 2^{k-i}$ which is always even.

Now consider the construction of the DNF of a Boolean function (with bias P) defined by such a good set. Suppose that in the recursive construction of the good set one begins by assigning 1s to the vertices of a j-cube ($j < k$). The first clause of the DNF is then just the AND (product) of all the $k - j$ literals involved to fill the vertices of that j-cube. If the next step of the recursive construction of the good set consists in assigning 1s to a i-cube ($i < j$), the second clause of the DNF will be the product of all $k - i$ previous literals. We can thus iteratively construct the DNF for the BF represented by the given good set.

Since the vertices get filled by 0s or 1s hierarchically from a j-cube to $j-1$-cube, after filling the 1-cube, we are left with another 1-cube to be filled. When output values of the vertices of this last 1-cube are to be fixed, both vertices have to be set to the same output value since P is even. Thus they will either contribute a clause with $k-1$ variables to the DNF expression (if the output values are set to 1) or they will not contribute any clause (if the output values are set to 0). Importantly, the variable which is missing in this clause is not present in any of the other clauses, therefore making that BF ineffective in that input. In constructing such a function, there will be at most $k-1$ variables in the Boolean expression. This implies that the resulting function has a Boolean complexity strictly less than k. See Fig. S7 for a visual proof of the above argument.

8. Average sensitivity of the network

The average sensitivity of the network is calculated by taking the mean of the average sensitivity over all nodes of the network. To determine the consequences of using different types of BFs in a network, we keep its structure (list of inputs to each node) but assign to each node a random function belonging to a particular type of BF (for example EF or CF), and compute the average sensitivity of the resulting Boolean model. For each biological network and a particular type of BF, we repeat the above procedure 1000 times and store the sampled data points. We performed this for all 88 models in our reference biological dataset using a broad range of BFs such as: EF, EUF, CF, ECF, NCF, RoF, non-NCF RoF. Finally, we plot the distribution for the obtained data points as a violin plot (see Fig. 5 in main text). Note that for the biological case there are only 88 data points corresponding to 88 networks or models, whereas in all other cases there are 88000 data points, as we sample 1000 data points for each type of BF per network. The computer programs to check the type of a BF and generate it is available at: `https://github.com/asamallab/MCBF`. Outlined below is the procedure used to generate random k-input BFs in each of the types mentioned above.

Effective functions (EF)

Choose a random integer between 0 and 2^{2^k} and convert the integer to its binary vector representation and check if the resulting BF is effective. If not, repeat the procedure till an EF is obtained.

Effective and unate functions (EUF)

Up to $k = 6$ inputs, all the UFs which are effective can be generated, hence a random choice from this list returns an EUF. If $k > 6$, a random partition of k is generated such that each element of the partition is a number less than or equal to 6. In other words, $k = k_1 + k_2 + k_3 + \ldots$ such that $k_i \leq 6$. A random EUF with k_1 variables is generated and combined with a random EUF with k_2 variables by either an AND or OR logic function. This is repeated till all elements of the partition are covered. For example, if $k = 10$, then an acceptable partition is $(2, 5, 3)$ and the EUF which is generated is $(EUF(2) \odot EUF(5)) \odot EUF(3)$ where $\odot$ is AND or OR (which is also chosen randomly for each occurrence). Since generating the UFs with greater than 6 inputs is computationally expensive, we resort to the heuristic algorithm provided above. The functions obtained using this heuristic may not give a uniform distribution over all EUFs.

Canalyzing functions (CF)

We implement the algorithm provided in the software BoolNet [25] to generate random CFs. Generate a random integer between 0 and 2^{2^k} and convert it to a binary vector. This is a random BF. If the function is not canalzying, choose one of the k inputs randomly and also choose a random canalyzing input value (0 or 1). Set the outputs corresponding those 2^{k-2} entries of the binary vector to 0 or 1 (also chosen randomly). Thus the generated function is guaranteed to be canalyzing in at least one input.

Effective and Canalyzing functions (ECF)

Generate a CF based on the procedure given above and check if the resulting BF is effective. If not, repeat the procedure till an EF is obtained.

Nested canalyzing functions (NCF)

We leverage the fact that for each bias, there is exactly one NCF upto isomorphisms. Hence, given k, randomly choose an odd bias between 1 and 2^{k-1}, say P. For bias P, the NCF is generated by setting the first P bits of the output binary vector to 1 and the remaining $2^k - P$ bits to 0. Note that since the average sensitivity is invariant under change of signs in the inputs, one can simply calculate the average sensitivity of any of the isomorphic forms of a NCF with bias P.

Read-once functions (RoF)

Since all the representative RoFs can be generated for $k \leq 10$, a RoF can be chosen randomly from such a list of representative RoFs. In case $k > 10$, we partition k into two parts such that $k = k_1 + k_2$, where $k_1 \leq 10$ and $k_2 \leq 10$. A randomly chosen k_1 input RoF is then combined with a randomly chosen k_2 input RoF by either an AND or OR operator which is also chosen randomly.

non-NCF Read-once functions (non-NCF RoF)

For $k \geq 4$, generate a random RoF and check if it is a NCF. If so, repeat the procedure till a non-NCF RoF is generated. In case a node has less than 4 inputs, random NCFs are assigned to them as there are no non-NCF RoF for $k < 4$.

Estimating the overlap between the distributions of average sensitivities for various types of BFs and the biological case

To estimate the extent of overlap between the distribution of network average sensitivities corresponding to a particular type of BF and the biological case, we compute the fraction of data points (of the distribution of the BF we are interested in) which are outliers when considering the biological distribution. The outlying regions are defined via the 5% of data points which fall on any one side of the biological distribution (one-sided test) or 2.5% of data points on either side (two-sided test) of the distribution. Note that if at the 2.5% (or 5%) threshold we get an average sensitivity value for which there are multiple data points, then it may be that only some of these data points may fall in that 2.5%. If so, then that value of average sensitivity is assigned a probability equal to the number of occurrences in the outlier divided by the total number of data points having that average sensitivity (in the biological distribution). Thus when counting the number of data points (in the distribution of some type of BF) falling in the outliers of the biological distribution, only a fraction (equal to the probability) of those data points having the threshold value of average sensitivity are counted as outliers. It is clear that the larger the fraction of data points that are outlier to the biological distribution, the more distant that distribution is from the biological case. From the data in Table S10 for the two-sided test, we can arrange various BFs based on their increasing proximity to the biological distribution in the following manner: EF $<$ ECF $<$ EUF $<$ CF $<$ non-NCF RoF $<$ RoF $<$ NCF.

9. Results from the repeat analyses after discarding the ineffective inputs to BFs in the reference biological dataset

In our reference biological dataset of 2687 BFs from 88 models, there are 63 ineffective BFs (see Table S6). Such ineffective BFs in the reference dataset are likely reconstruction errors in the model, and a possible way to mitigate any influence of these ineffective functions on the results from our analyses is by considering the truncated BF without the ineffective inputs. That is, for all of the 63 ineffective BFs in the reference dataset, we discard the ineffective inputs and consider the corresponding truncated effective BF. For instance, if a k-input BF has j ineffective inputs (where $k > j$), then the effective number of inputs in the BF is $k_{eff} = k - j$, which is also equal to the number of inputs in the truncated EF.

To confirm that the conclusions of this study are not affected by these ineffective BFs, we repeated our analyses (including relative abundance of biologically meaningful BFs and associated statistical tests, and distributions of network average sensitivities for the 88 models) by considering a modified reference biological dataset of 2687 BFs wherein each of the 63 ineffective BFs are replaced by their corresponding truncated EFs (see Fig. S8(a), Table S11). These results are reported in Figs. S8 and S9, and Tables S11, S12, S13, S14, S15, S16 and S17. From these additional figures and tables in the supplementary material, it is evident that all the conclusions we reach using the 2687 BFs (including the ineffective functions) in the reference biological dataset, remain unchanged when ineffective functions are replaced by their corresponding truncated EFs with k_{eff} effective inputs in the modified dataset.

SUPPLEMENTARY FIGURES

(a) **Truth Table**

x_4	x_3	x_2	x_1	$f(\mathbf{x})$
0	0	0	0	0
0	0	0	1	0
0	0	1	0	0
0	0	1	1	1
0	1	0	0	0
0	1	0	1	0
0	1	1	0	0
0	1	1	1	1
1	0	0	0	0
1	0	0	1	0
1	0	1	0	0
1	0	1	1	1
1	1	0	0	1
1	1	0	1	1
1	1	1	0	1
1	1	1	1	1

(b) **Ordered binary vector**

0
0
0
1
0
0
0
1
0
0
0
1
1
1
1
1

(c) **Boolean Expression**

Full Disjunctive Normal Form (DNF)

$$f(x) = \bar{x}_4\bar{x}_3x_2x_1 + \bar{x}_4x_3x_2x_1 + x_4\bar{x}_3x_2x_1 + x_4x_3\bar{x}_2\bar{x}_1 + x_4x_3\bar{x}_2x_1 + x_4x_3x_2\bar{x}_1 + x_4x_3x_2x_1$$

Factorized form

$$f(x) = x_1x_2 + x_3x_4$$

(d) **Boolean Hypercube**

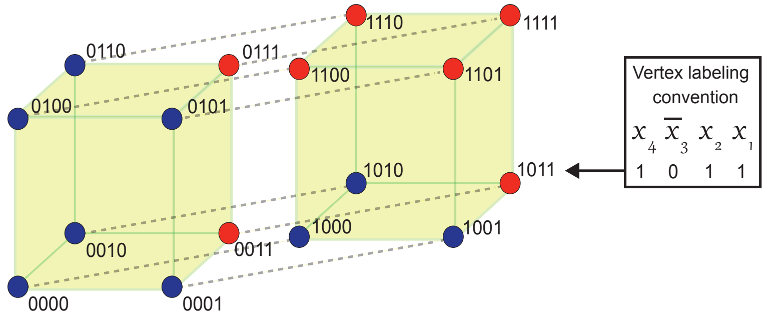

Fig. S1. Illustration of four different representations of Boolean functions (BFs) using an example. (a) Truth table. **(b)** Ordered binary vector, which is the output column of the truth table, but now taken as a k-dimensional vector. **(c)** Boolean expression. Multiple types of Boolean expressions are possible. We have shown for illustration the BF in the truth table in its Full Disjunctive Normal Form (DNF) and it's minimum equivalent expression. In the Full DNF, each row of the truth table whose output value is 1 is represented as a product of k literals, each one corresponding to one input bit. If an input bit is 0, it is represented by a negated literal, otherwise, by a positive one. **(d)** Colored Boolean Hypercube. Each vertex corresponds to one row of the truth table. The position of the vertex is defined by the inputs and the color of the vertex by the output corresponding to that input. The color blue is used for the output value "0" and the color red for the output value "1". The edges between any two blue (0s) vertices constitute E_{00} while the edges between any two red (1s) vertices constitute E_{11}. The edges between a blue (0) vertex and a red (1) vertex constitute E_{01}.

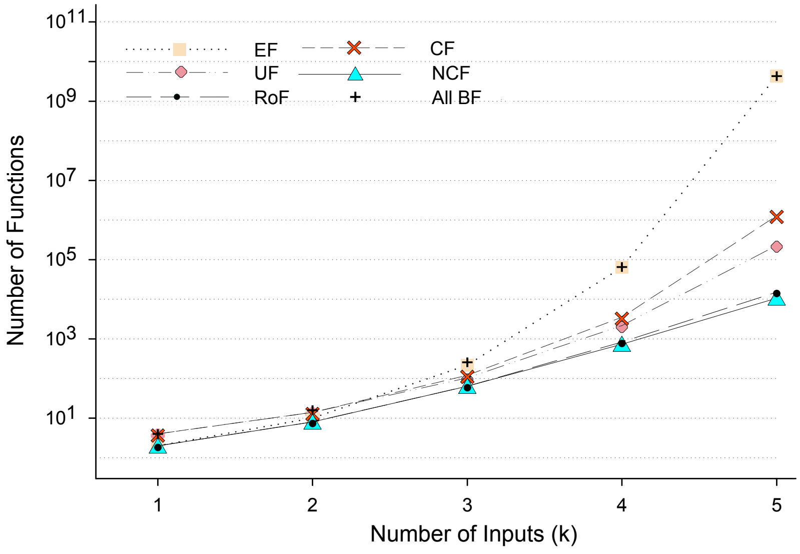

Fig. S2. The number of Boolean functions (BFs) for each biologically meaningful type, at a given number of inputs $k \leq 5$. Here, EF corresponds to effective functions, UF to unate functions (all sign combinations), CF to canalyzing functions, NCF to nested canalyzing functions, RoF to read-once functions, and "All BF" to all 2^{2^k} possible functions with k inputs.

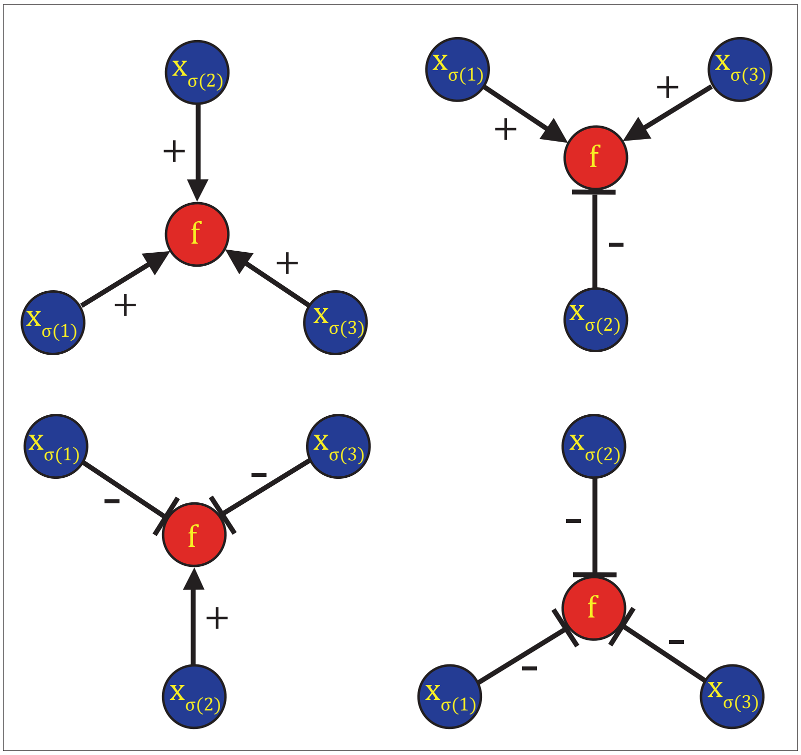

Fig. S3. Schematic figure depicting the four possible *sign* combinations for the $k = 3$ inputs to a node (gene) f. Each input or regulatory interaction to the node f can be an activator or inhibitor which are shown as '+' or '-', respectively. The 3 inputs to the node f are labeled by variables $x_{\sigma(1)}$, $x_{\sigma(2)}$ or $x_{\sigma(3)}$, without repetition of any label. σ is the permutation of the set $\{1, 2, 3\}$, and $\sigma(i)$ represents the ith element of a given permutation.

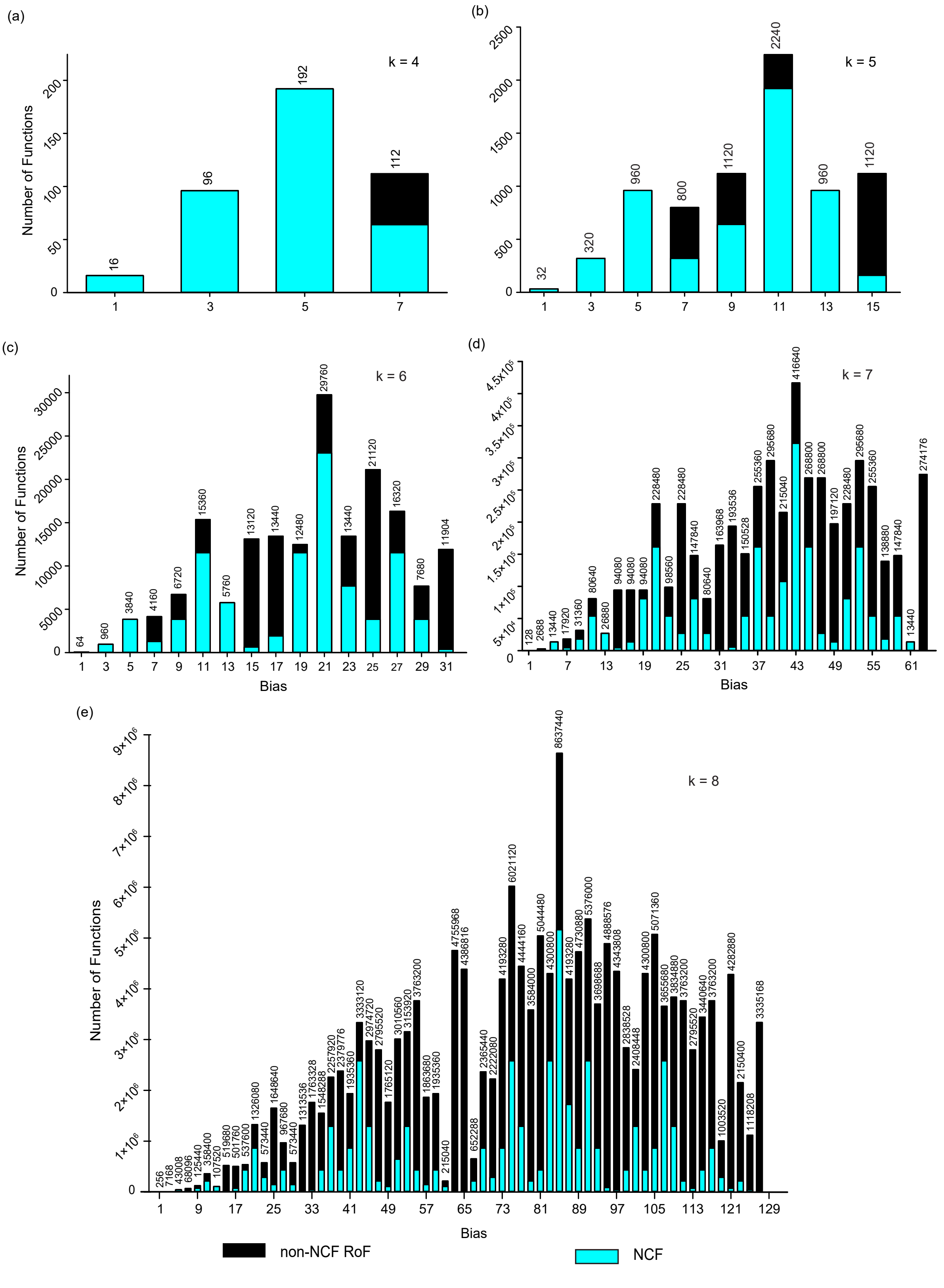

Fig. S4. Frequency distribution of read-once functions (RoFs) across different bias P for functions with **(a)** $k = 4$, **(b)** $k = 5$, **(c)** $k = 6$, **(d)** $k = 7$, and **(e)** $k = 8$ inputs. For each bar, we display the number of RoFs with that bias value. The figure also gives the frequency distribution of nested canalyzing functions (NCFs), which are a subset of RoFs. Due to the *complementarity* property of Boolean functions, the distribution is symmetric about the bias value 2^{k-1} for a given k, and therefore, we display only the first half of the distribution, from 0 to 2^{k-1} in each case.

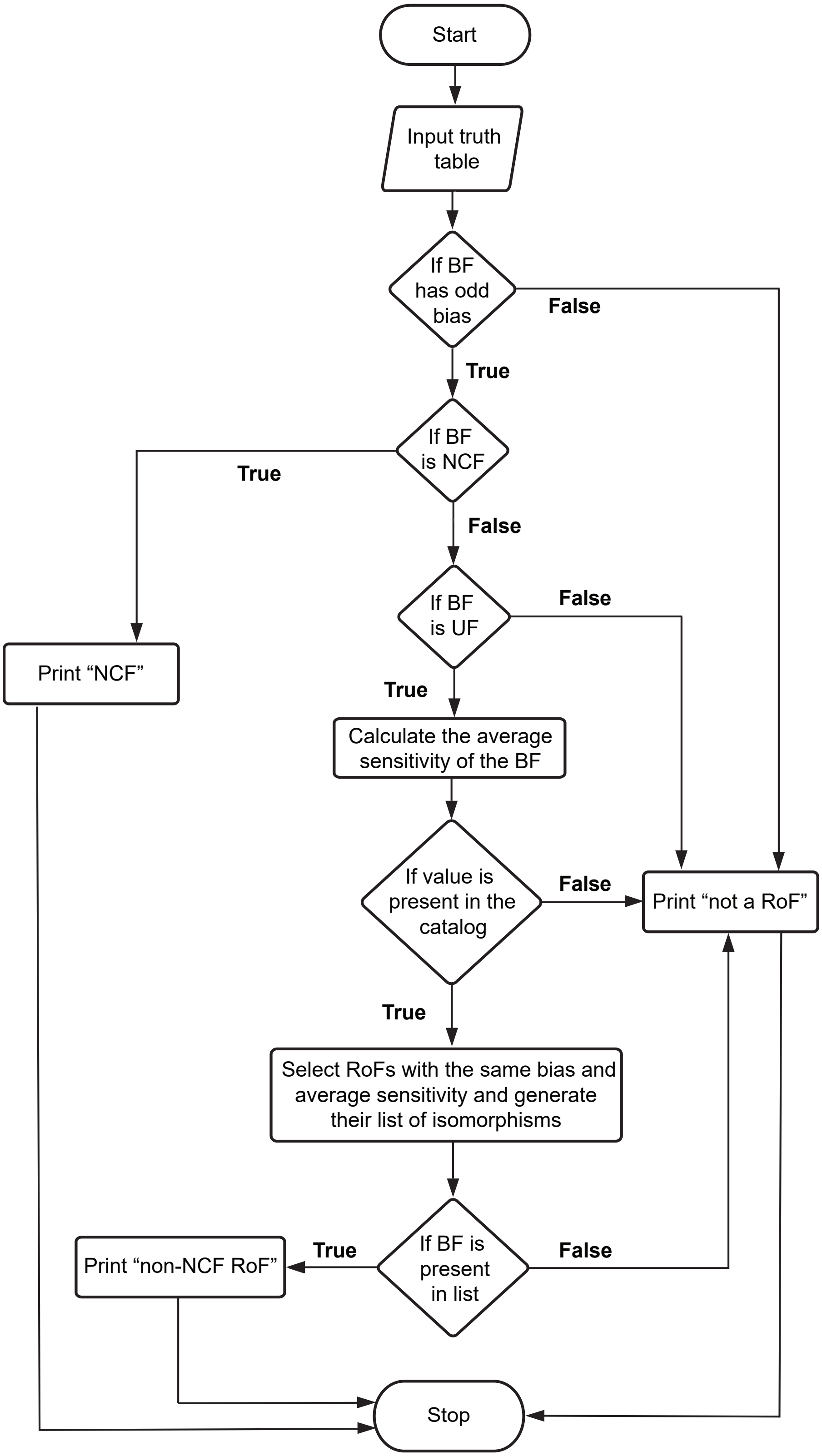

Fig. S5. Flowchat describing our program to check whether a Boolean function (BF) with $k \leq 10$ inputs entered by an user, is a read-once function (RoF). The program can also distinguish between a nested canalyzing function (NCF) and a 'non-NCF RoF'.

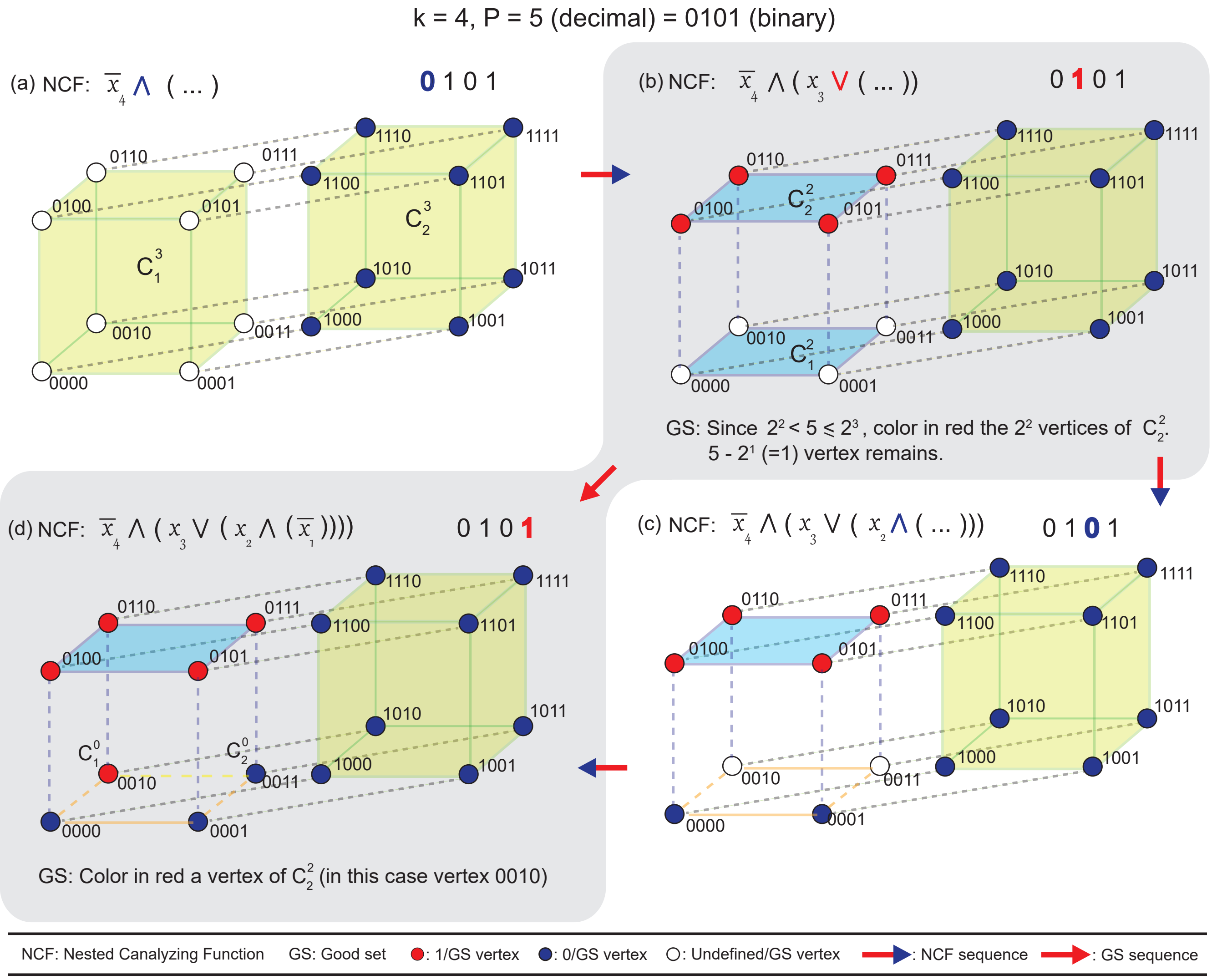

Fig. S6. 'Good set' (GS) for P vertices where P is odd on a k-dimensional hypercube is equivalent to a Nested Canalyzing Function (NCF) in that $k[P]$ set. In parts (b) and (d) shaded in grey, we show the recursive construction of a GS for $P = 5$ vertices in a 4-dimensional hypercube by coloring it's vertices red, and in parts (a), (b), (c) and (d), we show the equivalence of that GS of 5 vertices to a NCF with bias 5. The vertices of the hypercube are labeled in the order x_4, x_3, x_2, x_1 wherein x_i is 0 or 1. Here, C_1^j and C_2^j denote the two vertex disjoint j-dimensional hypercubes of the $(j+1)$-dimensional hypercube. The 'active' bit in each part (a), (b), (c) and (d) is the colored bit in the binary representation of 5 in that part. **(a)** The vertices with $x_4 = 1$ are canalyzed to the output value 0. The active bit in this step is 0 and as a result the $\wedge$ operator follows the literal $\overline{x}_4$. **(b)** Since $P = 5$ lies between 2^2 and 2^3, 2^2 vertices of either C_1^2 or C_2^2 (here, C_2^2) form part of the GS. This leaves $5 - 4 = 1$ vertex to be colored red to complete the GS. This choice of 4 vertices in C_2^2 for the GS leads to the canalyzation of vertices labeled $x_4 = 0$ and $x_3 = 1$ to the output value 1. The active bit in this step is 1 and as a result the $\vee$ operator follows the literal x_3. **(c)** The vertices with $x_4 = 0$, $x_3 = 0$ and $x_2 = 0$ are canalyzed to the output value 0. The active bit in this step is 0 and as a result the $\wedge$ operator follows the literal x_2. **(d)** For the last step, any vertex in C_1^2 can be colored to complete the 5 vertices in GS, and we color the vertex 0010. The vertex with $x_4 = 0$, $x_3 = 0$, $x_2 = 1$ and $x_1 = 0$ is canalyzed to the output value 1, and the one remaining vertex is set to output value 0.

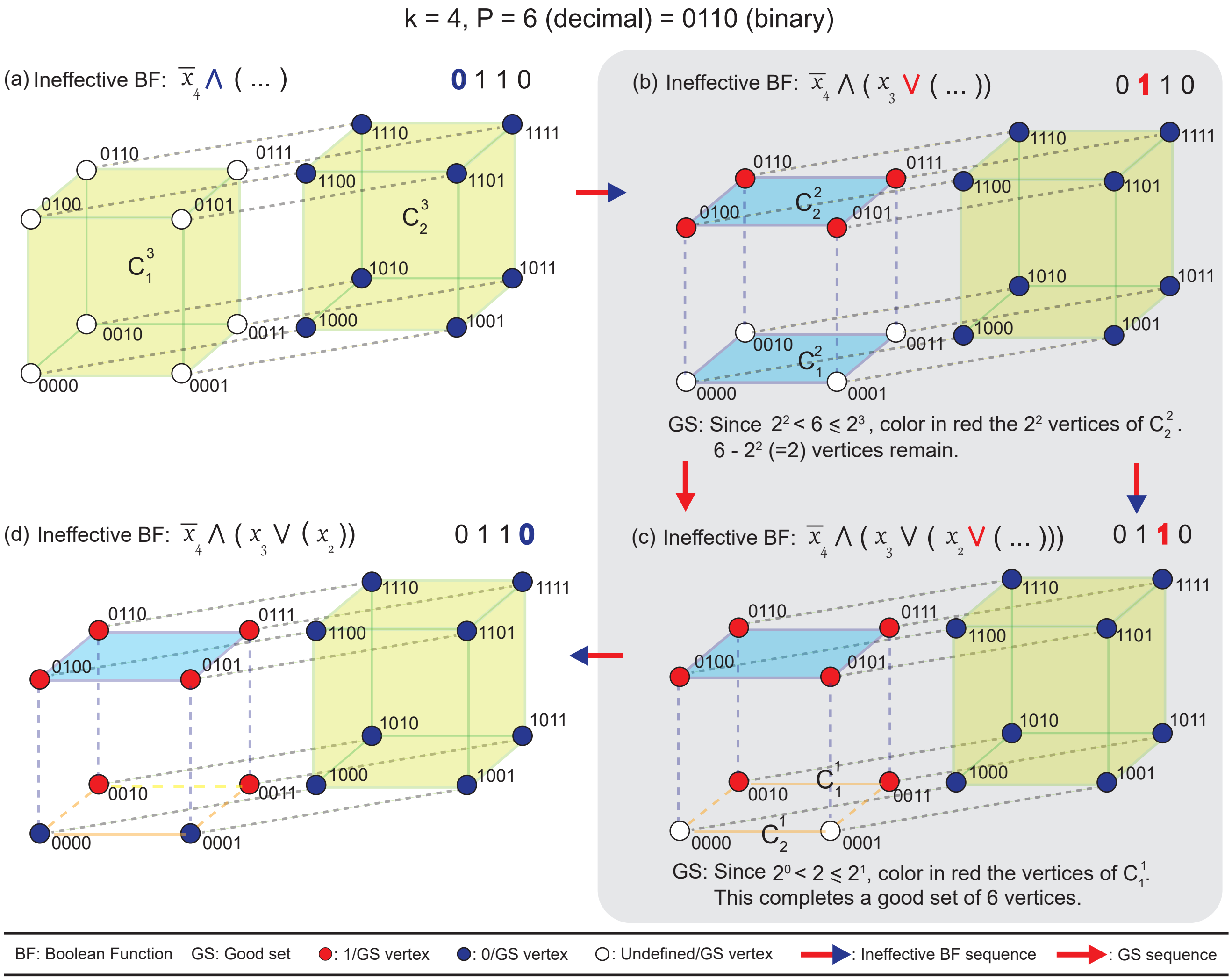

Fig. S7. 'Good set' (GS) for P vertices where P is even on a k-dimensional hypercube is equivalent to an ineffective Boolean Function (BF) in that $k[P]$ set. In parts (b) and (c) shaded in grey, we show the recursive construction of a GS for $P = 6$ vertices in a 4-dimensional hypercube by coloring it's vertices red, and in parts (a), (b), (c) and (d), we show the equivalence of that GS with 6 vertices to an ineffective BF with bias 6. The vertices of the hypercube are labeled in the order x_4, x_3, x_2, x_1 wherein x_i is 0 or 1. Here, C_1^j and C_2^j denote the two vertex disjoint j-dimensional hypercubes of the $(j+1)$-dimensional hypercube. The 'active' bit in each part (a), (b), (c) and (d) is the colored bit in the binary representation of 6 in that part. **(a)** The vertices with $x_4 = 1$ are set to the output value 0. The active bit in this step is 0 and as a result the $\wedge$ operator follows the literal $\overline{x}_4$. **(b)** Since $P = 6$ lies between 2^2 and 2^3, 2^2 vertices of either C_1^2 or C_2^2 (here, C_2^2) form part of the GS. This leaves $6 - 4 = 2$ vertices to be colored to complete the GS. This choice of 4 vertices in C_2^2 for the GS leads to setting the output value of vertices labeled $x_4 = 0$ and $x_3 = 1$ to 1. The active bit in this step is 1 and as a result the $\vee$ operator follows the literal x_3. **(c)** Since the remaining 2 vertices lies between 2^1 and 2^2, 2^1 vertices of either C_1^1 or C_2^1 (here, C_1^1) form part of the GS. This completes the GS of 6 vertices. This choice of 2 vertices in C_1^1 for the GS leads to setting the output value of vertices labeled $x_4 = 0$, $x_3 = 0$, and $x_2 = 1$ to 1. The active bit in this step is 1 and as a result the $\vee$ operator follows the literal x_2. **(d)** Since the two remaining undefined vertices take the same value 0, the variable x_1 does not appear in the expression of the BF. Thus, the BF corresponding to GS with 6 vertices is ineffective.

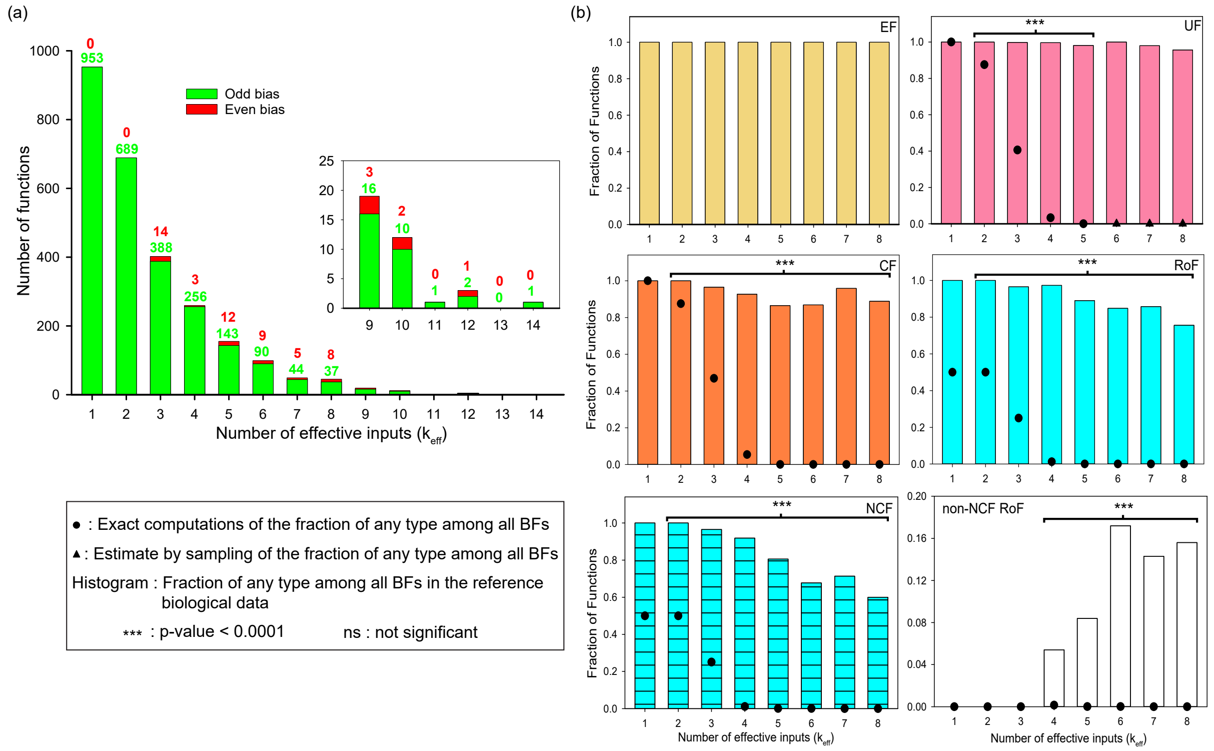

Fig. S8. (a) The in-degree distribution for nodes in the modified reference biological dataset after discarding the ineffective inputs in ineffective functions. Here, k_{eff} is the number of effective inputs after stripping a BF of its ineffective inputs. **(b)** The plots show the abundance and statistical significance of the biologically meaningful BFs for $k_{eff} \leq 8$ in the modified dataset. The dot symbols which appear to coincide with the x-axis are very small non-zero numbers (except for non-NCF RoFs with $k = 1, 2, 3$). We do not show the p-values of the EF case since checking its statistical significance is not meaningful as all BFs are forced to be effective in the modified dataset. The raw data associated with these plots along with results from the statistical test for over-representation are included in Tables S11, S12 and S13.

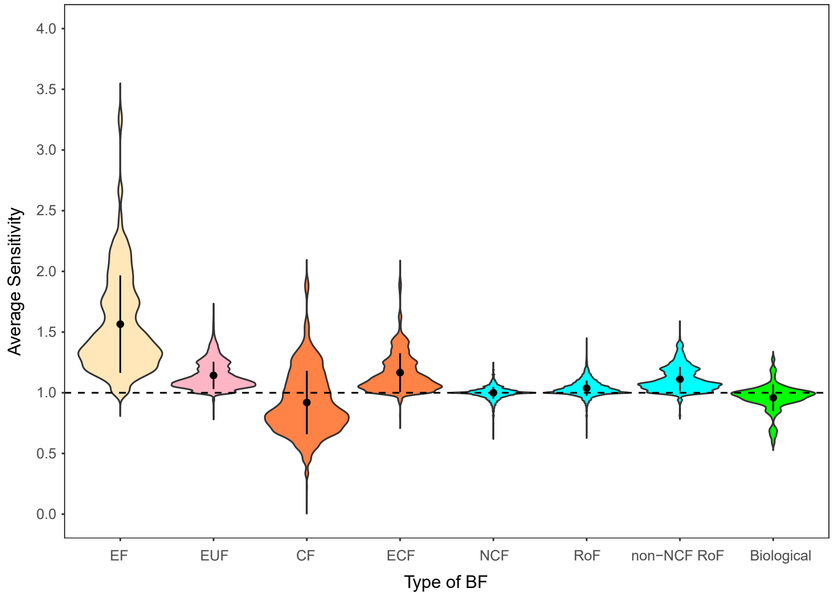

Fig. S9. Distribution of the network average sensitivity when using the list of (effective) inputs from biological models but enforcing different types of BFs to the nodes, namely effective functions (EF), effective and unate functions (EUF), canalyzing functions (CF), effective and canalyzing functions (ECF), nested canalyzing functions (NCF), read-once functions (RoF) and non-NCF RoFs. For this computation, we start with the modified reference biological dataset wherein all ineffective inputs to nodes are discarded in each of the 88 networks or models. The right-most case is the distribution when using the actual BFs in the biological models. This plot has been generated by keeping the maximum width of each of the 'violins' fixed.

SUPPLEMENTARY TABLES

Table S1. The number of Boolean functions (BFs) belonging to the different types, at a given number of inputs $k \leq 5$. Here, EF corresponds to effective functions, UF to unate functions (all sign combinations), CF to canalyzing functions, EUF to effective and unate functions, ECF to effective and canalyzing functions, UCF to unate and canalyzing functions, EUCF to effective, unate and canalyzing functions. In addition, the table lists the total number of BFs for each k.

k	Types of BFs							
	**All**	**EF**	**UF**	**CF**	**EUF**	**ECF**	**UCF**	**EUCF**
1	4	2	4	4	2	2	4	2
2	16	10	14	14	8	8	14	8
3	256	218	104	120	72	88	96	64
4	65536	64594	2170	3514	1824	3104	1178	864
5	4294967296	4294642034	230540	1292276	220608	1275784	36796	31744

Table S2. The fraction of Boolean functions (BFs) belonging to the different types, at a given number of inputs $k \leq 5$. Here, EF corresponds to effective functions, UF to unate functions (all sign combinations), CF to canalyzing functions, EUF to effective and unate functions, ECF to effective and canalyzing functions, UCF to unate and canalyzing functions, EUCF to effective, unate and canalyzing functions.

k	Types of BFs						
	**EF**	**UF**	**CF**	**EUF**	**ECF**	**UCF**	**EUCF**
1	0.500	1.000	1.000	0.500	0.500	1.000	0.500
2	0.625	0.875	0.875	0.500	0.500	0.875	0.500
3	0.852	0.406	0.469	0.281	0.344	0.375	0.250
4	0.986	0.033	0.054	0.028	0.047	0.018	0.013
5	1.000	5.37×10^{-5}	3.01×10^{-4}	5.14×10^{-5}	2.97×10^{-4}	8.57×10^{-6}	7.39×10^{-6}

Table S3. Parity distribution of biologically meaningful types of Boolean functions (BFs) for a given number of inputs k. The fraction of even parity functions of a particular type of BF is calculated with respect to the total number of functions of that BF type. Here, EF corresponds to effective functions, UF to unate functions (all sign combinations), CF to canalyzing functions, EUF to effective and unate functions, ECF to effective and canalyzing functions, UCF to unate and canalyzing functions, EUCF to effective, unate and canalyzing functions. Note that entries labeled '-' are those which could not be computed due to inadequate computational resources.

k	Number of even parity functions								Fraction of even parity functions							
	**All**	**EF**	**UF**	**CF**	**EUF**	**ECF**	**UCF**	**EUCF**	**All**	**EF**	**UF**	**CF**	**EUF**	**ECF**	**UCF**	**EUCF**
1	2	0	2	2	0	0	2	0	0.5	0	0.5	0.5	0	0	0.5	0
2	8	2	6	6	0	0	6	0	0.5	0.2	0.429	0.429	0	0	0.429	0
3	128	90	40	56	8	24	32	0	0.5	0.413	0.385	0.467	0.111	0.273	0.333	0
4	32768	31826	922	1754	576	1344	442	128	0.5	0.493	0.425	0.499	0.316	0.433	0.375	0.148
5	2147483648	–	110348	646132	100416	629640	15932	10880	0.5	–	0.479	0.5	0.455	0.494	0.433	0.343

Table S4. The number of effective and unate functions (EUFs) at a given number of inputs $k \leq 5$ for different combinations of activators and inhibitors. The table also gives the fraction of EUFs that have even bias for different sign combinations. These numbers have been obtained via exhaustive computational enumeration of EUFs.

k	**Activators**	**Inhibitors**	**EUFs**		
			**Total**	**Even bias**	**Fraction with Even bias**
1	1	0	1	0	0
	0	1	1	0	0
2	2	0	2	0	0
	1	1	4	0	0
	0	2	2	0	0
3	3	0	9	1	0.111
	2	1	27	3	0.111
	1	2	27	3	0.111
	0	3	9	1	0.111
4	4	0	114	36	0.316
	3	1	456	144	0.316
	2	2	684	216	0.316
	1	3	456	144	0.316
	0	4	114	36	0.316
5	5	0	6894	3138	0.455
	4	1	34470	15690	0.455
	3	2	68940	31380	0.455
	2	3	68940	31380	0.455
	1	4	34470	15690	0.455
	0	5	6894	3138	0.455

Table S5. The number of Nested Canalyzing functions (NCFs) and Read-once functions (RoFs), and their fraction among all Boolean functions (BFs) for a given number of inputs k. In addition, the table lists the fraction of NCFs among RoFs for a given number of inputs k.

k	**Number of**		**Fraction of**		**Fraction of NCFs among RoFs**
	**NCFs**	**RoFs**	**NCFs**	**RoFs**	
1	2	2	0.500	0.500	1.000
2	8	8	0.500	0.500	1.000
3	64	64	0.250	0.250	1.000
4	736	832	0.011	0.013	0.885
5	10624	15104	2.47×10^{-6}	3.52×10^{-6}	0.703
6	183936	352256	9.97×10^{-15}	1.91×10^{-14}	0.522
7	3715072	10037248	1.09×10^{-32}	2.95×10^{-32}	0.370
8	85755372	337936384	7.41×10^{-70}	2.92×10^{-69}	0.254
9	2226939904	13126565888	1.66×10^{-145}	9.79×10^{-145}	0.170
10	64255903744	577818263552	3.57×10^{-298}	3.21×10^{-297}	0.111

Table S6. Number of different types of biologically meaningful Boolean functions (BFs) in the reference biological dataset. Here, k is the number of inputs, 'All' is the total number of BFs for a given number of inputs, EF corresponds to effective functions, UF to unate functions (all sign combinations), CF to canalyzing functions, EUF to effective and unate functions, ECF to effective and canalyzing functions, UCF to unate and canalyzing functions, EUCF to effective, unate and canalyzing functions, NCF to Nested Canalyzing functions and RoF to Read-once functions.

k	Types of BFs									
	**All**	**EF**	**UF**	**CF**	**EUF**	**ECF**	**UCF**	**EUCF**	**NCF**	**RoF**
1	934	934	934	934	934	934	934	934	934	934
2	687	671	687	687	671	671	687	671	671	671
3	412	392	411	398	391	378	398	378	378	378
4	258	251	257	239	250	232	239	232	230	244
5	156	149	153	136	146	129	135	128	120	133
6	107	98	107	93	98	85	93	85	67	83
7	51	48	50	49	47	46	48	45	34	41
8	45	45	43	40	43	40	38	38	27	34
9	19	19	18	17	18	17	17	17	7	16
10	13	12	13	11	12	10	11	10	3	6
11	1	1	1	1	1	1	1	1	0	0
12	3	3	3	3	3	3	3	3	2	2
13	0	0	0	0	0	0	0	0	0	0
14	1	1	1	1	1	1	1	1	1	1

Table S7. Fraction of different types of biologically meaningful Boolean functions (BFs) in the reference biological dataset. Here, k is the number of inputs, EF corresponds to effective functions, UF to unate functions (all sign combinations), CF to canalyzing functions, EUF to effective and unate functions, ECF to effective and canalyzing functions, UCF to unate and canalyzing functions, EUCF to effective, unate and canalyzing functions, NCF to Nested Canalyzing functions and RoF to Read-once functions.

k	Types of BFs								
	**EF**	**UF**	**CF**	**EUF**	**ECF**	**UCF**	**EUCF**	**NCF**	**RoF**
1	1	1	1	1	1	1	1	1	1
2	0.977	1	1	0.977	0.977	1	0.977	0.977	0.977
3	0.951	0.998	0.966	0.949	0.917	0.966	0.917	0.917	0.917
4	0.973	0.996	0.926	0.969	0.899	0.926	0.899	0.891	0.946
5	0.955	0.981	0.872	0.936	0.827	0.865	0.821	0.769	0.853
6	0.916	1	0.869	0.916	0.794	0.869	0.794	0.626	0.776
7	0.941	0.980	0.961	0.922	0.902	0.941	0.882	0.667	0.804
8	1	0.956	0.889	0.956	0.889	0.844	0.844	0.6	0.756
9	1	0.947	0.895	0.947	0.895	0.895	0.895	0.368	0.842
10	0.923	1	0.846	0.923	0.769	0.846	0.769	0.231	0.462
11	1	1	1	1	1	1	1	0	0
12	1	1	1	1	1	1	1	0.667	0.667
13	-	-	-	-	-	-	-	-	-
14	1	1	1	1	1	1	1	1	1

Table S8. p-value tests for statistical enrichments of the different types of Boolean functions (BFs) in the reference biological dataset. A low p-value indicates that the corresponding type of BF is enriched in the reference biological dataset when compared to the ensemble of all BFs. For $k > 2$ when the p-value shown is 0, it was smaller than what we could measure, and when the p-value shown is 1, its deviation from 1 was smaller than we could measure. Here, EF corresponds to effective functions, UF to unate functions (all sign combinations), CF to canalyzing functions, NCF to Nested Canalyzing functions and RoF to Read-once functions.

k	**Odd bias**	**EF**	**UF**	**CF**	**NCF**	**RoF**
2	3.742×10^{-177}	6.75×10^{-114}	0	0	3.74×10^{-177}	3.74×10^{-177}
3	6.253×10^{-76}	2.44×10^{-11}	6.65×10^{-162}	1.83×10^{-111}	3.09×10^{-184}	3.09×10^{-184}
4	2.714×10^{-62}	0.919	0	8.86×10^{-279}	0	0
5	2.718×10^{-25}	1	0	0	0	0
6	1.753×10^{-13}	1	0	0	0	0
7	6.058×10^{-08}	1	0	0	0	0
8	1.561×10^{-06}	1	0	0	0	0

Table S9. The relative enrichment ratios E_R for the RoFs and NCFs in the ensemble of odd bias BFs, EFs and UFs. These ratios indicate the extent of the over-representation of such functions in the reference biological dataset. $E_R > 1$ suggests that there is indeed an enrichment of RoFs and NCFs within the EFs, UFs and CFs in the reference biological dataset when compared to that expected in the ensemble of all EFs, UFs and CFs.

k	E_R **for RoF in:**			E_R **for NCF in:**		
	**Odd bias**	**EF**	**UF**	**Odd bias**	**EF**	**UF**
1	1	1	2	1	1	2
2	1.0	1.25	1.75	1.0	1.25	1.75
3	2.0	3.284	1.567	2.0	3.284	1.567
4	38.770	75.483	2.536	41.279	80.367	2.700
5	1.37×10^5	2.54×10^5	13.633	1.76×10^5	3.25×10^5	17.47

Table S10. The percentage of data points that fall outside the 95% confidence interval of the biological case in the distribution of network average sensitivities when using the list of inputs from biological models but enforcing different types of BFs to the nodes, namely effective functions (EF), effective and unate functions (EUF), canalyzing functions (CF), effective and canalyzing functions (ECF), nested canalyzing functions (NCF), read-once functions (RoF) and non-NCF RoFs, The distribution of network avererage sensitivities is shown in Fig. 5 and data for both one-sided tests and two-sided tests are provided here.

**Type of BF**	**One-sided (upper 5%)**	**One-sided (lower 5%)**	**Two-sided (2.5% on either side)**
EF	92.61	0.0	87.27
EUF	39.35	0.0	30.69
CF	20.38	16.61	26.75
ECF	43.13	0.0	35.05
NCF	0.75	0.0	0.04
RoF	5.92	0.0	2.29
non-NCF RoF	32.4	0.0	22.4

Table S11. Number of different types of biologically meaningful BFs in the modified reference biological dataset. Here, k_{eff} is the number of effective inputs after stripping a BF of its ineffective inputs. 'All' is the total number of BFs for a given number of effective inputs, EF corresponds to effective functions, UF to unate functions (all sign combinations), CF to canalyzing functions, EUF to effective and unate functions, ECF to effective and canalyzing functions, UCF to unate and canalyzing functions, EUCF to effective, unate and canalyzing functions, NCF to Nested Canalyzing functions and RoF to Read-once functions.

k_{eff}	Types of BFs									
	**All**	**EF**	**UF**	**CF**	**EUF**	**ECF**	**UCF**	**EUCF**	**NCF**	**RoF**
1	953	953	953	953	953	953	953	953	953	953
2	689	689	689	689	689	689	689	689	689	689
3	402	402	401	388	401	388	388	388	388	388
4	259	259	258	240	258	240	240	240	238	252
5	155	155	152	134	152	134	133	133	125	138
6	99	99	99	86	99	86	86	86	67	84
7	49	49	48	47	48	47	46	46	35	42
8	45	45	43	40	43	40	38	38	27	34
9	19	19	18	17	18	17	17	17	7	16
10	12	12	12	10	12	10	10	10	3	6
11	1	1	1	1	1	1	1	1	0	0
12	3	3	3	3	3	3	3	3	2	2
14	1	1	1	1	1	1	1	1	1	1

Table S12. Fraction of different types of biologically meaningful BFs in the modified reference biological dataset. Here, k_{eff} is the number of effective inputs after stripping a BF of its ineffective inputs. EF corresponds to effective functions, UF to unate functions (all sign combinations), CF to canalyzing functions, EUF to effective and unate functions, ECF to effective and canalyzing functions, UCF to unate and canalyzing functions, EUCF to effective, unate and canalyzing functions, NCF to Nested Canalyzing functions and RoF to Read-once functions.

k_{eff}	Types of BFs								
	**EF**	**UF**	**CF**	**EUF**	**ECF**	**UCF**	**EUCF**	**NCF**	**RoF**
1	1	1	1	1	1	1	1	1	1
2	1	1	1	1	1	1	1	1	1
3	1	0.998	0.965	0.998	0.965	0.965	0.965	0.965	0.965
4	1	0.996	0.927	0.996	0.927	0.927	0.927	0.919	0.973
5	1	0.981	0.865	0.981	0.865	0.858	0.858	0.806	0.890
6	1	1	0.869	1	0.869	0.869	0.869	0.677	0.848
7	1	0.980	0.959	0.980	0.959	0.939	0.939	0.714	0.857
8	1	0.956	0.889	0.956	0.889	0.844	0.844	0.600	0.756
9	1	0.947	0.895	0.947	0.895	0.895	0.895	0.368	0.842
10	1	1	0.833	1	0.833	0.833	0.833	0.25	0.5
11	1	1	1	1	1	1	1	0	0
12	1	1	1	1	1	1	1	0.667	0.667
14	1	1	1	1	1	1	1	1	1

Table S13. p-value tests for statistical enrichments of the different types of BFs in the modified reference biological dataset. Here, k_{eff} is the number of effective inputs after stripping a BF of its ineffective inputs. A low p-value indicates that the corresponding type of BF is enriched in the modified reference biological dataset when compared to the ensemble of all BFs. For $k_{eff} > 2$ when the p-value shown is 0, it was smaller than what we could measure, and when the p-value shown is 1, its deviation from 1 was smaller than we could measure. Here, EF corresponds to effective functions, UF to unate functions (all sign combinations), CF to canalyzing functions, NCF to Nested Canalyzing functions and RoF to Read-once functions.

k_{eff}	**Odd bias**	**UF**	**CF**	**NCF**	**RoF**
2	0	0	0	0	0
3	9.46×10^{-98}	5.43×10^{-158}	2.59×10^{-108}	1.43×10^{-212}	1.43×10^{-212}
4	3.63×10^{-74}	0	5.10×10^{-280}	0	0
5	5.12×10^{-31}	0	0	0	0
6	2.95×10^{-19}	0	0	0	0
7	4.11×10^{-10}	0	0	0	0
8	1.56×10^{-6}	0	0	0	0

Table S14. Fractions of functions that are RoFs, non-NCF RoFs or NCFs, in the space of all $2^{2^{k_{eff}}}$ BFs (f_0) or in the modified reference biological dataset (f_1). $E(= f_1/f_0)$ is the enrichment ratio; it indicates the extent of the over-representation of such functions in the modified reference dataset. Over-representation is highest for NCFs but clearly non-NCF RoFs are also highly over-represented. Computations are reported for functions with $k_{eff} \leq 8$ inputs.

k_{eff}	RoF			non-NCF RoF			NCF		
	f_0	f_1	E	f_0	f_1	E	f_0	f_1	E
1	0.5	1.0	2.0	0	0	-	0.5	1.0	2.0
2	0.5	1.0	2.0	0.0	0.0	-	0.5	1.0	2.0
3	0.25	1.0	4.0	0.0	0.0	-	0.25	1.0	4.0
4	0.0127	0.965	76.012	0.001	0.0	0.0	0.011	0.965	85.927
5	3.517×10^{-06}	0.973	2.77×10^5	1.04×10^{-06}	0.054	5.18×10^4	2.47×10^{-06}	0.919	3.71×10^5
6	1.909×10^{-14}	0.89	4.66×10^{13}	9.12×10^{-15}	0.084	9.21×10^{12}	9.97×10^{-15}	0.806	8.08×10^{13}
7	2.950×10^{-32}	0.848	2.87×10^{31}	1.86×10^{-32}	0.172	9.26×10^{30}	1.092×10^{-32}	0.677	6.20×10^{31}
8	2.918×10^{-69}	0.857	2.94×10^{68}	2.18×10^{-69}	0.143	6.57×10^{67}	7.404×10^{-70}	0.714	9.64×10^{68}

Table S15. The relative enrichment ratios E_R for the RoFs and NCFs in the ensemble of odd bias BFs, EFs and UFs in the modified dataset. Here, k_{eff} is the number of inputs after stripping a BF of its ineffective inputs. These enrichment ratios indicate the extent of the over-representation of such functions in the modified reference biological dataset. $E_R > 1$ suggests that there is indeed an enrichment of RoFs and NCFs within the EFs, UFs and CFs in the modified reference biological dataset when compared to that expected in the ensemble of all EFs, UFs and CFs.

k_{eff}	E_R for RoF in:			E_R for NCF in:		
	**Odd bias**	**EF**	**UF**	**Odd bias**	**EF**	**UF**
1	1.0	1.0	2	1.0	1.0	2
2	1.0	1.25	1.75	1.0	1.25	1.75
3	2.0	3.40625	1.625	2.0	3.40625	1.625
4	39.385	74.920	2.517	44.522	84.692	2.845
5	1.40×10^5	2.77×10^5	14.851	1.88×10^5	3.71×10^5	19.94

Table S16. The relative enrichment ratio E_R of the NCFs in the CFs and RoFs in the modified dataset. $f_{s,0}/f_0$ denotes the fractions of functions that are NCFs in the space of all CFs or RoFs and $f_{s,1}/f_1$, the equivalent fraction in the modified reference biological dataset. Here, k_{eff} is the number of inputs after stripping a BF of its ineffective inputs. $E_R = (f_{s,1}/f_1)/(f_{s,0}/f_0)$ denotes the enrichment ratio and it indicates the extent of the over-representation of such functions in the modified reference dataset. Computations are reported for BFs with $k_{eff} \leq 8$ inputs. The low p-values indicate that there is an enrichment of NCFs within the CFs and RoFs in the modified reference dataset when compared to that expected in the ensemble of all CFs and RoFs.

k_{eff}	NCF in CF				NCF in RoF			
	$f_{s,0}/f_0$	$f_{s,1}/f_1$	E_R	p-value	$f_{s,0}/f_0$	$f_{s,1}/f_1$	E_R	p-value
2	0.571	1.0	1.75	0	1.0	1.0	1.0	0
3	0.533	1.0	1.875	0	1.0	1.0	1.0	0
4	0.209	1.0	4.774	1.04×10^{-160}	0.885	1.0	1.130	3.87×10^{-4}
5	0.008	0.991	120.588	3.73×10^{-251}	0.703	0.944	1.343	2.02×10^{-9}
6	1.78×10^{-6}	0.932	5.22×10^5	0	0.522	0.906	1.734	4.06×10^{-8}
7	7.19×10^{-15}	0.779	1.08×10^{14}	0	0.370	0.798	2.157	1.04×10^{-10}
8	7.87×10^{-33}	0.744	9.45×10^{31}	0	0.254	0.833	3.283	5.26×10^{-12}

Table S17. The percentage of data points that fall outside the 95% confidence interval of the modified biological case in the distribution of network average sensitivities when using the list of inputs from biological models but enforcing different types of BFs to the nodes, namely effective functions (EF), effective and unate functions (EUF), canalyzing functions (CF), effective and canalyzing functions (ECF), nested canalyzing functions (NCF), read-once functions (RoF) and non-NCF RoFs, The distribution of network avererage sensitivities is shown in Fig. S9 and data for both one-sided tests and two-sided tests are provided here. From the data for the two-sided test, we can arrange various BFs based on their increasing proximity to the biological distribution in the following manner: EF < ECF < EUF < CF < non-NCF RoF < RoF < NCF.

**Type of BF**	**One-sided (upper 5%)**	**One-sided (lower 5%)**	**Two-sided (2.5% on either side)**
EF	91.85	0.0	86.26
EUF	38.68	0.0	29.84
CF	19.7	17.31	26.72
ECF	42.5	0.0	34.51
NCF	0.75	0.01	0.05
RoF	5.53	0.0	2.06
non-NCF RoF	31.88	0.0	22.05

References

1. S. A. Kauffman. *The origins of order: self-organization and selection in evolution*. Oxford University Press, New York, 1993.
2. M. Anthony. *Discrete Mathematics of Neural Networks*. Society for Industrial and Applied Mathematics, Philadelphia, 2001.
3. J. Aracena. Maximum Number of Fixed Points in Regulatory Boolean Networks. *Bulletin of Mathematical Biology*, 70(5): 1398, 2008.
4. C. Kadelka, J. Kuipers, and R. Laubenbacher. The influence of canalization on the robustness of Boolean networks. *Physica D: Nonlinear Phenomena*, 353:39–47, 2017.
5. Z. Szallasi and S. Liang. Modeling the normal and neoplastic cell cycle with 'realistic Boolean genetic networks': Their application for understanding carcinogenesis and assessing therapeutic strategies. In *Pacific Symposium on Biocomputing*, volume 3, pages 66–76. Citeseer, 1998.
6. S. A. Kauffman, C. Peterson, B. Samuelsson, and C. Troein. Random Boolean network models and the yeast transcriptional network. *Proceedings of the National Academy of Sciences*, 100(25):14796–14799, 2003.
7. S. Nikolajewa, M. Friedel, and T. Wilhelm. Boolean networks with biologically relevant rules show ordered behavior. *BioSystems*, 90(1):40–47, 2007.
8. J. P. Hayes. The Fanout Structure of Switching Functions. *Journal of the Association for Computing Machinery*, 22(4): 551–571, 1975.
9. J. Feldman. Minimization of Boolean complexity in human concept learning. *Nature*, 407(6804):630–633, 2000.
10. T. Helikar, B. Kowal, S. McClenathan, M. Bruckner, T. Rowley, A. Madrahimov, B. Wicks, M. Shrestha, K. Limbu, and J. A. Rogers. The Cell Collective: Toward an open and collaborative approach to systems biology. *BMC Systems Biology*, 6(1): 1–14, 2012.
11. A. G. Gonzalez, A. Naldi, L. Sanchez, D. Thieffry, and C. Chaouiya. GINsim: A software suite for the qualitative modelling, simulation and analysis of regulatory networks. *BioSystems*, 84(2):91–100, 2006.
12. C. Li, M. Donizelli, N. Rodriguez, H. Dharuri, L. Endler, V. Chelliah, L. Li, E. He, A. Henry, M. I. Stefan, J. L. Snoep, M. Hucka, N. L. Novère, and C. Laibe. BioModels Database: An enhanced, curated and annotated resource for published quantitative kinetic models. *BMC Systems Biology*, 4(1):1–14, 2010.
13. E. Guberman, H. Sherief, and E. R. Regan. Boolean model of anchorage dependence and contact inhibition points to coordinated inhibition but semi-independent induction of proliferation and migration. *Computational and Structural Biotechnology Journal*, 18:2145–2165, 2020.
14. A. Singh, J. M. Nascimento, S. Kowar, H. Busch, and M. Boerries. Boolean approach to signalling pathway modelling in HGF-induced keratinocyte migration. *Bioinformatics*, 28(18):i495–i501, 2012.
15. K. S. Song, J. K. Seong, K. C. Chung, W. J. Lee, C. H. Kim, K. N. Cho, D. D. Kang, J. S. Koo, and J. H. Yoon. Induction of MUC8 gene expression by interleukin-1β is mediated by a sequential ERK MAPK/RSK1/CREB cascade pathway in human airway epithelial cells. *Journal of Biological Chemistry*, 278(37):34890–34896, 2003.
16. F. Herrmann, A. Groß, D. Zhou, H. A. Kestler, and M. Kühl. A Boolean Model of the Cardiac Gene Regulatory Network Determining First and Second Heart Field Identity. *PLOS ONE*, 7(10):e46798, 2012.
17. A. Méndez and L. Mendoza. A Network Model to Describe the Terminal Differentiation of B Cells. *PLOS Computational Biology*, 12(1):e1004696, 2016.
18. E. Remy, S. Rebouissou, C. Chaouiya, A. Zinovyev, F. Radvanyi, and L. Calzone. A Modeling Approach to Explain Mutually Exclusive and Co-Occurring Genetic Alterations in Bladder Tumorigenesis. *Cancer Research*, 75(19):4042–4052, 2015.
19. S. Von der Heyde, C. Bender, F. Henjes, J. Sonntag, U. Korf, and T. Beissbarth. Boolean ErbB network reconstructions and perturbation simulations reveal individual drug response in different breast cancer cell lines. *BMC Systems Biology*, 8(1):1–22, 2014.
20. A. Saadatpour, R. S. Wang, A. Liao, X. Liu, T. P. Loughran, I. Albert, and R. Albert. Dynamical and Structural Analysis of a T Cell Survival Network Identifies Novel Candidate Therapeutic Targets for Large Granular Lymphocyte Leukemia. *PLOS Computational Biology*, 7(11):e1002267, 2011.
21. E. Azpeitia, M. Benítez, I. Vega, C. Villarreal, and E. R. Alvarez-Buylla. Single-cell and coupled grn models of cell patterning in the *Arabidopsis thaliana* root stem cell niche. *BMC Systems Biology*, 4(1):1–19, 2010.
22. X. Gan and R. Albert. Analysis of a dynamic model of guard cell signaling reveals the stability of signal propagation. *BMC Systems Biology*, 10(1):1–14, 2016.
23. W. Just, I. Shmulevich, and J. Konvalina. The number and probability of canalizing functions. *Physica D: Nonlinear Phenomena*, 197(3):211–221, 2004.
24. Sasao and Kinoshita. On the number of fanout-free functions and unate cascade functions. *IEEE Transactions on Computers*, C-28(1):66–72, 1979.
25. C. Müssel, M. Hopfensitz, and H. A. Kestler. BoolNet – an R package for generation, reconstruction and analysis of Boolean networks. *Bioinformatics*, 26(10):1378–1380, 2010.
